# Hypoxia-induced BTN3A2 promotes glioma progression and chemoresistance via AKT/SP1/RAD51-mediated DNA damage

**DOI:** 10.1038/s41419-026-08729-7

**Published:** 2026-04-11

**Authors:** Zhixing Xu, Shanrui Pu, Jin Wu, Shengjing Yuan, Xiaobing Huang, Jintao Tian, Xingchang Li, Bohu Liu, Xinglong Yu, Jinxi Zhao, Fengcai shi, Xi Chen, Jun Pu

**Affiliations:** 1 Department of Neurosurgery, The second Affiliated Hospital of Kunming Medical University, Kunming, China; 2 Department of Neurosurgery, Pu’er People’s Hospital, Kunming University of Science and Technology Affiliated Hospital, Yunnan, China; 3 School of Biosciences, University of Birmingham, Birmingham, UK; 4 Department of Respiratory and Critical Care Medicine, Pu’er People’s Hospital, Kunming University of Science and Technology Affiliated Hospital, Yunnan, China

**Keywords:** CNS cancer, CNS cancer, Tumour biomarkers

## Abstract

Glioma remains a highly aggressive malignancy with frequent recurrence and resistance to radiotherapy and chemotherapy. BTN3A2 is a multifunctional regulatory protein originally implicated in γδ T-cell–mediated immune responses, yet its tumor-intrinsic role and mechanistic relevance in glioma are poorly defined. Here, BTN3A2 expression and prognostic associations were assessed in TCGA and CGGA cohorts and further validated by immunohistochemistry on tissue microarrays. Functional studies using lentivirus-mediated BTN3A2 knockdown demonstrated that BTN3A2 promotes glioma cell proliferation, migration, and invasion, and its depletion increases TMZ sensitivity in vitro and in vivo. Mechanistically, integrated RNA-seq, CUT&Tag, and promoter luciferase assays identified BTN3A2 as a hypoxia-responsive gene directly transcriptionally activated by HIF-1α. BTN3A2 subsequently enhanced DNA damage repair capacity through activation of the AKT/SP1/RAD51 axis, thereby contributing to TMZ resistance. Collectively, these findings establish BTN3A2 as a hypoxia-driven, cell-intrinsic mediator of glioma progression and chemoresistance, highlighting its potential value as a prognostic biomarker and therapeutic vulnerability.

## Introduction

Glioma, particularly glioblastoma (GBM), remains the most prevalent and lethal primary malignancy of the central nervous system [1]. Despite aggressive multimodal treatment consisting of maximal safe surgical resection followed by radiotherapy and temozolomide (TMZ) chemotherapy, clinical outcomes remain dismal, with a median overall survival of approximately 15 months and a 5-year survival rate of less than 8% [2–4]. A major cause of treatment failure is the inevitable development of chemoresistance, which markedly limits the long-term efficacy of TMZ [5]. As an alkylating agent, TMZ exerts its cytotoxic effects primarily through the formation of O^6-methylguanine lesions, ultimately leading to DNA double-strand breaks (DSBs) and apoptosis [6]. Consequently, activation of DNA damage response (DDR) pathways—particularly homologous recombination (HR) repair—enables glioma cells to efficiently resolve lethal DNA lesions, thereby promoting cell survival and therapeutic resistance [7].

Importantly, chemoresistance in glioma is not solely driven by intrinsic genetic alterations but is dynamically shaped by the tumor microenvironment (TME). Hypoxia, a hallmark of rapidly proliferating gliomas, profoundly influences tumor cell fate by reprogramming stress-adaptive and survival pathways [8]. Hypoxic signaling has been shown to promote dedifferentiation, enhance resistance to cytotoxic therapies, and modulate DNA repair capacity in cancer cells [9]. Hypoxia-inducible factor-1α (HIF-1α) serves as a master regulator of these adaptive responses; however, the downstream molecular effectors that connect hypoxia signaling to enhanced DDR activity and sustained glioma cell survival remain incompletely understood.

The BTN3A family in humans comprises three subtypes: BTN3A1, BTN3A2, and BTN3A3, characterized by two extracellular domains (IgV and IgC), a transmembrane domain, a cytoplasmic region containing a juxtamembrane (JM) region, and a B30.2 intracellular domain [10–12]. Early studies on BTN3A family members have predominantly focused on their role in immune regulation, particularly their capacity to sense intracellular phosphoantigens and transmit metabolic signals to Vγ9Vδ2 T cells, thereby modulating γδ T cell activation, proliferation, and anti-tumor cytotoxicity [13–15]. These discoveries have unveiled a novel mechanism of tumor immune surveillance and immune evasion, establishing BTN3A molecules as promising targets in cancer immunotherapy. Accordingly, BTN3A-targeted therapeutic strategies have progressed to clinical development, exemplified by ITC01, a γδ T cell–activating antibody currently undergoing Phase I/II clinical trials [16]. Nevertheless, emerging evidence suggests that BTN3A family members may also exert cell-intrinsic functions, independent of their role in immune regulation.

Among the BTN3A family, BTN3A2 has recently been implicated in tumor progression through non-immune mechanisms. Aberrant expression of BTN3A2 has been reported in solid tumors such as breast cancer and pancreatic ductal adenocarcinoma, where it modulates cancer cell stemness and proliferation [17, 18]. These findings suggest that BTN3A2 may play a role in fundamental cellular processes that regulate tumor cell survival. However, the expression pattern, biological role, and mechanistic contribution of BTN3A2 in glioma—particularly in the context of hypoxia adaptation and DNA damage repair—remain largely unexplored.

In this study, we investigated the clinical relevance and functional role of BTN3A2 in glioma progression and therapeutic resistance. We demonstrate that BTN3A2 is significantly upregulated in high-grade gliomas and correlates with adverse molecular features and poor patient prognosis. Functional analyses reveal that BTN3A2 promotes glioma cell proliferation, migration, and invasion, while conferring resistance to TMZ-induced genotoxic stress. Mechanistically, we identify BTN3A2 as a transcriptional target of HIF-1α under hypoxic conditions and show that BTN3A2 enhances DNA damage repair capacity and cell survival through activating a p-AKT/SP1/RAD51-associated regulatory axis. Collectively, our findings uncover a previously unrecognized, cell-intrinsic role of BTN3A2 in linking hypoxia signaling to DNA damage response, providing new insights into glioma cell survival and chemoresistance.

## Materials and methods

### Bioinformatics analysis

Gene expression profiles and clinical data for glioma patients were retrieved from the TCGA database (https://portal.gdc.cancer.gov/) and normalized using the R package “limma.” Likewise, standardized BTN3A2 expression data across multiple cancer types were obtained from the OncoDB database (https://oncodb.org/cgi-bin/genomic_normal_expression_search.cgi). Additionally, standardized mRNA expression data (mRNA-array_325) and associated clinical information were downloaded from the Chinese Glioma Genome Atlas (CGGA) (http://www.cgga.org.cn) and further normalized. Following the 2021 World Health Organization classification criteria for central nervous system tumors, low-grade gliomas were defined as WHO grades I–II, and high-grade gliomas as WHO grades III–IV [19].

### Immunohistochemistry

Glioma tissue microarrays (HBraG159Su01) with clinicopathological annotation were purchased from Shanghai Outdo Biotechnology Co., Ltd. Detailed clinical information for all patients is provided in Table S1. Tissue sections were incubated at 65 °C for 2 h, then dewaxed and rehydrated. Antigen retrieval was performed by heating the sections in 0.01 M citrate buffer (pH6.0) at 95 °C. Following blocking with normal goat serum, sections were incubated overnight at 4 °C with primary anti-BTN3A2 antibody (1:100, ab118051, Abcam, USA). The next day, sections were incubated with secondary antibodies for 1 h and visualized using DAB staining (Maixin Biotechnology Development Co., Ltd., Fuzhou, China). After counterstaining with hematoxylin, the sections were dehydrated and mounted. Evaluation was performed independently by two observers. BTN3A2 staining intensity was scored semiquantitatively as: 0 (no staining), 1 (weak), 2 (moderate), and 3 (strong). The percentage of positive tumor cells was scored as: 0 (0–5%), 1 (5–25%), 2 (26–50%), 3 (51–75%), and 4 (76–100%). The final immunoreactivity score was calculated by multiplying the two scores. Samples were then categorized into a BTN3A2 low expression group (score <6) and a BTN3A2 high expression group (score 6–12).

### Cells, cell culture and transfection

Normal human astrocyte (NHA) cells were maintained in commercial astrocyte culture medium (No.1801, ScienCell) supplemented with 2% fetal bovine serum (FBS; ScienCell), 1% astrocyte growth supplement (AGS; ScienCell), and 1% penicillin–streptomycin (P/S; ScienCell). LN229, U87, and SF295 glioma cell lines were cultured in DMEM (Hyclone) supplemented with 10% FBS (Gibco, 10270-106) and 1% penicillin–streptomycin in a humidified incubator at 37°C with 5% CO₂. Lentiviral vectors encoding human BTN3A2-specific shRNA (target sequence 1: 5′-TGGAAAGTACTTGTGTTATTT-3′; target sequence 2: 5′-CAGATGGAGTGGGCCTATATG-3′) or a non-targeting scramble control (5′-TTCTCCGAACGTGTCACGT-3′) were obtained from GenePharma (Shanghai, China). U87 and SF295 cells were transduced with lentivirus for 72 h, followed by selection with puromycin (2 μg/mL) for 7days. To rigorously exclude off-target effects, this experiment used HIF-1α-specific siRNA and scrambled siRNA controls, which were purchased from GenePharma (Suzhou, China). Two independent siRNAs targeting human HIF-1α were used: si-HIF-1α#1 (5′-CCAUGUGACCAUGAGGAAATT-3′) and si-HIF-1α#2 (5′-GGGAUAUGAGCCAGAAGAATT-3′). Cells seeded in 6-well plates were transfected using Lipofectamine 3000 (Invitrogen, Carlsbad, CA, USA) according to the manufacturer’s instructions. After 48 h, cells were harvested for downstream assays. For hypoxic treatment in vitro, cells were incubated under 1% O_2_ conditions. To assess temozolomide (TMZ) resistance in vitro, U87 and SF295 cells were exposed to various concentrations of TMZ (Proteintech, Cat No: CM00537, China) for 24 h.

### RNA isolation and RT-PCR

RNA extraction and RT-PCR Total RNA of SF295 and U87 cell lines was extracted using TRIzol reagent (Invitrogen). cDNA was synthesized utilizing the Prime Script RT reagent kit with gDNA Eraser (Kogen, Tokyo, Japan), and real-time quantitative PCR was conducted employing SYBR Green II Mixture (TaKaRa) in accordance with the manufacturer’s instructions. mRNA expression was quantified via the relative Ct method (ΔΔCt), employing β-actin as the internal control. The designated primer pairs were as follows: β-actin primers (upstream primer, 5′-AAGTGTGACGTGGACATCCGC-3′; reverse primer, 5′-AAGTGTGACGTGGACATCCGC-3′); BTN3A2 primers (upstream primer: 5′-ATGAAAATGGCAAGTTCCCTGGCTT-3′; reverse primer, 5′-TCAGCTTCACCCACC-3′).

### Western blot

Total proteins were extracted utilizing RIPA buffer (NO.P0013B, Bio-Tech Biotech Co., Ltd., China) supplemented with protease and phosphatase inhibitors (78442, Thermo Fisher Scientific, Carlsbad, CA). The lysate was subsequently centrifuged at 12,000 rpm for 15 min. The lysate was subjected to heating at 100 °C for 5 min and subsequently combined with the loading buffer. An equal quantity of protein was applied to 8–12% SDS-PAGE and subsequently transferred to nitrocellulose membranes. The PVDF membrane was subsequently blocked with 5% skim milk for 1 h and treated with the primary antibody at 4 °C overnight. The membrane was incubated with the secondary antibody (Proteintech, Wuhan, China, 1:5000) at room temperature in a light-protected setting for 1 h and subsequently developed. The Western blot analysis was conducted three times. The primary antibodies used are shown in Table S2.

### Clonogenic and Cell Counting Kit-8 (CCK8) Assay

1000 cells were counted and seeded in 6-well plates. The cells were grown in DMEM supplemented with 10% FBS, and the intervention group received temozolomide treatment for 24 h. Subsequently, cells were cultured in complete medium for approximately 14 days to allow colony formation. Colonies were fixed in 4% paraformaldehyde for 30 min at room temperature, stained with 0.01% crystal violet for 12 h, washed twice with phosphate-buffered saline (PBS), and then counted under a microscope. For the CCK-8 assay, 3000 cells per well were plated into 96-well plates in 100 μL DMEM containing 10% FBS. Cell viability was assessed using the CCK-8 kit (Bioss, Beijing, China) according to the manufacturer’s protocol. Briefly, 10 μL of CCK-8 reagent diluted in 100 μL of serum-free medium was added to each well and incubated at 37 °C for 2 h. Absorbance at 450 nm was measured using a microplate reader (Tecan, Mönnedorf, Switzerland). The proliferation rate was calculated as: (OD experimental − OD blank) / (OD control − OD blank) × 100%. All experiments were performed in triplicate to ensure reproducibility.

### Scratch assay and transwell assay

For the scratch assay, glioma cells were seeded into 6-well plates and cultured until reaching over 90% confluence. A uniform linear scratch was created using a sterile pipette tip, after which cells were maintained in serum-free DMEM. Wound closure was monitored and imaged, and the migration area was quantified using ImageJ software. For the Transwell assay, 4 × 10^4^ glioma cells were resuspended in serum-free medium and seeded into the upper chambers of Transwell inserts (Millipore). In the invasion assay, inserts were pre-coated with Matrigel, while migration assays were performed without Matrigel coating. The lower chambers were filled with 800 μL of DMEM containing 10% FBS. After 48 h incubation at 37 °C, cells on the upper membrane surface were removed, while cells on the lower surface were fixed with 4% paraformaldehyde for 15 min and stained with 0.1% crystal violet for 15 min before counting.

### Immunofluorescence staining

Cells were fixed in 4% paraformaldehyde for 15 min, permeabilized with 0.5% Triton X-100 in PBS for 10 min, and rinsed three times with PBS. Non-specific binding was blocked with 1% BSA at room temperature for 30 min. Subsequently, primary antibodies were applied and incubated overnight at 4 °C in a humid chamber. The following day, cells were incubated with fluorescently labeled secondary antibodies (Antigen, Wuhan, China) for 1 h at 37 °C in the dark. Slides were mounted with anti-fade mounting medium containing DAPI (P36962, Thermo Fisher Scientific, Carlsbad, CA) and visualized. For paraffin-embedded tissue sections, antigen retrieval was performed by microwave heating in retrieval buffer followed by cooling to room temperature. Endogenous horseradish peroxidase (HRP) activity was inhibited by incubation with a 3% hydrogen peroxide solution for 20 min. Sections were blocked with 10% normal goat serum for 30 min at room temperature, followed by incubation with primary antibodies overnight at 4 °C. Fluorescently labeled secondary antibodies were applied at 37 °C for 1 h in the dark. Sections were mounted with DAPI-containing mounting medium and imaged using confocal laser scanning microscopy.

### Flow cytometry analysis

Cells were inoculated in 6-well plates. Apoptosis of glioma cells was assessed using the ANNEXIN V-Alexa Fluor 647/PI Kit (CA1050, Beijing Solebo Technology Co., Ltd., China), while the cell cycle was evaluated with the ANNEXIN V-Alexa Fluor 647/PI Kit (C1052, Bio-Tech Biotech Co., Ltd., China). All procedures were executed in accordance with the manufacturer’s guidelines. The FACS flow cytometer (BD Biosciences, USA) was employed to assess apoptosis in the samples. The total apoptosis rate was determined by summing early and late apoptosis. The cell cycle distribution and its alterations were examined using flow cytometry following staining, and corresponding distribution diagrams and statistics charts were generated. All measurements were repeated three times.

### EdU incorporation assay

Cells were inoculated onto 24-well plates at a density of 1 × 10^5^ cells per well and incubated for 24 h. The CellLightTM-EdU imaging detection kit (Ruibo Biotech Co., Ltd., Guangzhou, China) was utilized for detection in accordance with the manufacturer’s guidelines.

### TUNEL assay

The DNA fragmentation of apoptotic cells in transplanted tumors was assessed using the In Situ Cell Death Detection Kit, following the manufacturer’s instructions (Roche). The sections were dewaxed at 60 °C on a heat block for 20 min and thereafter incubated in xylene for three intervals of five minutes each. Sections were rehydrated through graded ethanol washes and rinsed with PBS. After permeabilization with 0.1% Triton X-100 and treatment with Proteinase K, sections were incubated with TUNEL reaction mixture, followed by Converter-POD and DAB staining. Images were then captured and analyzed.

### CUT & tag sequencing

U87 cells underwent treatment with 1% O_2_ hypoxia for 12 h and were subsequently processed into fresh cell suspensions for experimentation following the standard CUT&Tag protocol. Briefly, cells were immobilized on magnetic beads conjugated with protein A/G and incubated with a primary antibody against HIF-1α (1:50; 36169S, Cell Signaling Technology, USA) or a normal rabbit IgG control (1:100; AF1935, R&D Systems) at 4 °C to detect the target transcription factor. Following PBS washing, a secondary antibody (transposase Tn5 complex with protein A/G fusion) was introduced to attach to the previously formed antibody complex, thereby facilitating the precise anchoring of the target genomic region. Upon the addition of Mg2+to activate Tn5 transposase, the transposase cleaved and ligated DNA next to the target site, resulting in the formation of library fragments suitable for sequencing. The DNA fragments placed into the adaptor were subsequently removed, and PCR amplified using particular primers to generate the final library for high-throughput sequencing. Sequencing was performed on an Illumina NovaSeq platform. Raw reads were filtered using FastQC and aligned to the human reference genome (hg38) using Bowtie2. Peaks were called using MACS2 with the IgG sample serving as the background control (*q*-value < 0.05). Downstream analyses, including motif enrichment and genomic distribution, were performed using HOMER and ChIPseeker.

### Luciferase reporter assay

The BTN3A2 promoter region (approx. 200 bp including the predicted HIF-1α binding site) and its mutant variant were cloned into the pGL3-basic reporter vector (BTN3A2-WT/MT). HIF-1α coding sequence (CDS) was cloned into the pcDNA3.1 vector, and constructs were sequence-verified. Cells co-transfected with these plasmids were assessed for luciferase activity using a dual-luciferase assay kit (JKR23008, Wuhan Jinkairui Bioengineering Co., Ltd., China), normalized to Renilla luciferase activity. All experiments were performed in triplicate.

### In vivo experiments

All animal procedures were approved by the Ethics Committee of the Second Affiliated Hospital of Kunming Medical University (Approval No. kyfey2023084) and followed the Guide for the Care and Use of Laboratory Animals. Male BALB/c nude mice (5 weeks old) were purchased from Beijing Vital River Laboratory Animal Technology Co., Ltd. and housed under specific pathogen-free (SPF) conditions.

### Orthotopic intracranial xenograft model

For the establishment of orthotopic glioma xenografts, 1 × 10^6^ luciferase-labeled glioma cells (NC or shBTN3A2) suspended in 10 μL PBS were stereotactically injected into the right striatum of anesthetized mice (coordinates relative to bregma: 2.0 mm lateral, 1.0 mm anterior, and 3.0 mm depth). Tumor growth was monitored weekly by bioluminescence imaging using an IVIS Spectrum imaging system following intraperitoneal injection of D-luciferin (150 mg/kg). Mice were randomized into four groups (*n* = 5 per group) : (1) NC, (2) NC + TMZ, (3) shBTN3A2, and (4) shBTN3A2 + TMZ. Temozolomide (30 mg/kg) was administered intraperitoneally daily for up to 21 days. Survival was recorded daily and analyzed using Kaplan-Meier survival curves. Animals were euthanized when they reached predefined humane endpoints or when a significant difference in tumor burden was observed between treatment groups.

### Subcutaneous xenograft model

For subcutaneous xenograft experiments, 1 × 10^7^ cells were injected subcutaneously into the axillary region. Starting on day 7, mice received intraperitoneal injections of TMZ (50 mg/kg) every three days. Tumors were assessed biweekly, and tumor volume was determined using the following formula: Total tumor volume (mm³) =0.5 × L×W², where L represents the long diameter and W denotes the short diameter. All mice were euthanized on the 20th day post-transplantation, and the tumor weights were recorded.

Different TMZ dosing regimens were applied in orthotopic and subcutaneous xenograft models to accommodate model-specific drug exposure and tolerability constraints, including differences in intracranial versus peripheral tumor microenvironments, in accordance with commonly adopted glioma xenograft protocols [20–22].

### Statistical analysis

Data were analyzed using SPSS 22.0 and GraphPad Prism 9.0. Normality and variance homogeneity were tested. Survival analyses were performed by the Kaplan-Meier method with the Log-rank test. Student’s t-test was used for two-group comparisons, and ANOVA for multiple-group comparisons. Significance thresholds were *p < 0.05, **p < 0.01, ***p < 0.001, ****p < 0.0001. All experiments were conducted independently at least three times.

## Results

### BTN3A2 is highly expressed in gliomas and correlated with prognosis

**Fig. 1:**
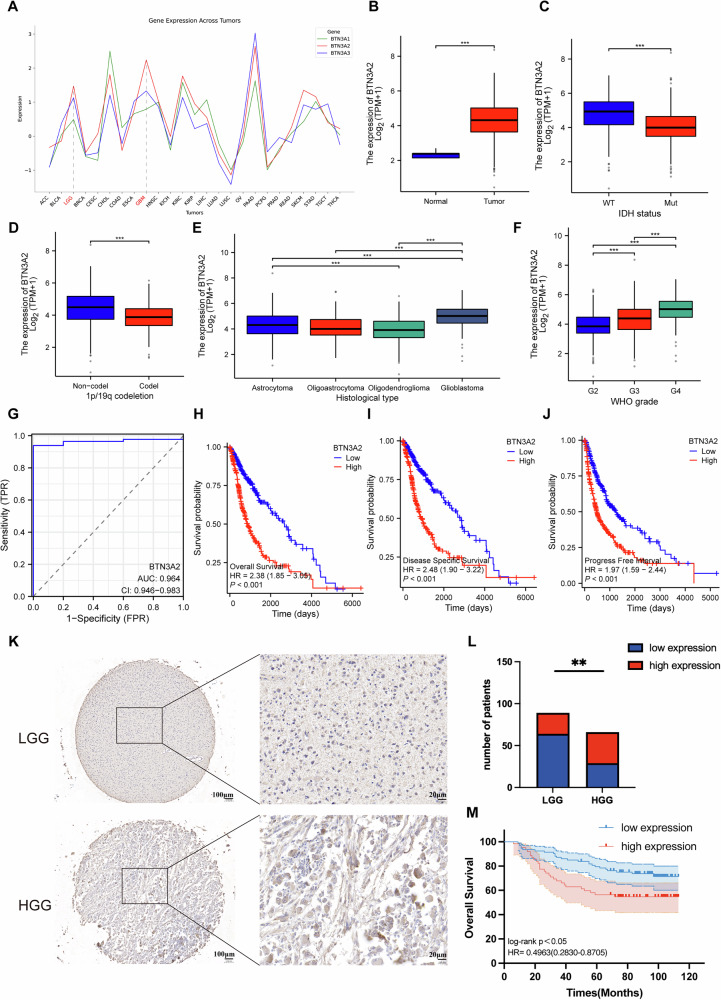
BTN3A2 is overexpressed in glioma and associates with prognosis in glioma. **A** mRNA expression levels of BTN3A1, BTN3A2, and BTN3A3 across pan-cancer datasets from the OncoDB. **B** Expression analysis of BTN3A2 in glioma versus normal brain tissues using TCGA and GTEx datasets. **C**, **D** BTN3A2 is enriched in IDH-wildtype and 1p/19q non-codeletion subgroups. **E**, **F** BTN3A2 expression in relation to molecular subtypes of glioma. **G** AUC values were estimated to assess the predictive ability of BTN3A2 expression. **H**, **J** Kaplan-Meier survival analyses of overall survival (OS), Disease specific survival(DSS)and progression-free survival (PFS) in glioma patients stratified by BTN3A2 expression levels in TCGA cohorts.**K** IHC images of BTN3A2 staining in glioma tissue microarray. Scale bar: 100 μm, 20 μm.**L**, **M** BTN3A2 high expression is associated with adverse clinical outcome by Kaplan-Meier plot analysis. *P < 0.05; **P < 0.01; ***P < 0.001.

This study utilized RNA-seq data of BTN3A from various cancers in the OncoDB database, revealing that the mRNA expression of BTN3A2 was markedly elevated in both low-grade and high-grade gliomas compared to other subtypes (Fig. 1A). We subsequently utilized the TCGA database to validate the elevated expression of BTN3A2 in gliomas and analyzed its expression across varying IDH1/2 statuses and 1p/19q co-deletion conditions. The expression of BTN3A2 was markedly elevated in IDH1/2 wild-type and 1p/19q non-co-deleted gliomas (Fig. 1B–D). Moreover, study of the TCGA and CGGA databases revealed considerable disparities in BTN3A2 expression across various glioma tissue types, with expression levels of BTN3A2 exhibiting a positive correlation with glioma grade (Fig. 1E, F, Fig. S1A–C). Receiver operating characteristic (ROC) curve analysis indicated that the area under the curve (AUC) for BTN3A2 in individuals with low-grade glioma was 0.964. Cox regression analysis, both univariate and multivariate, indicated that BTN3A2 serves as an independent prognostic risk factor for patients (Fig. 1G, Fig. S1D, E).

We endeavored to investigate the predictive significance of BTN3A2 in glioblastoma utilizing available datasets. BTN3A2 expression levels were dichotomized at the median, and survival outcomes were assessed using Kaplan-Meier analysis. Analysis of the TCGA, CGGA, and REMBRANDT datasets revealed that glioma patients with higher BTN3A2 expression exhibited significantly shorter overall survival compared to those with lower expression (Fig. 1H–J, Figure S1F–H). In addition, immunohistochemical staining—accounting for four off-target cases—was performed on tissue microarrays to determine BTN3A2 expression across high-grade and low-grade gliomas. As shown in Fig. 1K, L, BTN3A2 was primarily localized in the cytoplasm, with markedly higher expression in high-grade gliomas. The samples were categorized into high expression and low expression groups based on the average level of BTN3A2 expression. The expression of BTN3A2 was strongly associated with glioma grade and survival duration (Table 1). The Kaplan-Meier curve and survival analysis indicated that the overexpression of BTN3A2 is associated with a poor prognosis (Fig. 1M). The clinicopathological variables of all patients are shown in Table S1.

**Table 1:** The correlation of clinicopathological characteristics of glioma patients with BTN3A2 expression.

Characteristic	Expression of BTN3A2		*P* value
	Low	High	
**Age**			
≥45 (*n* = 69)	46	23	0.117
<45 (*n* = 86)	53	33	
**Gender**			
Female (*n* = 56)	37	19	0.633
Male (*n* = 99)	62	37	
**WHO grade**			
I (*n* = 17)	12	5	**0.006**
II (*n* = 72)	52	20	
III (*n* = 48)	26	22	
IV (*n* = 18)	9	9	
**Recurrence state**			
Recurrence (*n* = 85)	52	33	0.188
No recurrence (*n* = 70)	47	23	
**Overall survival**			
Death (*n* = 102)	30	23	**0.025**
Alive (*n* = 53)	69	33	

Bold values indicate statistical signifi cance (*P*＜0.05).

### BTN3A2 is a proto-oncogene that facilitates glioma survival both in vitro and in vivo

**Fig. 2:**
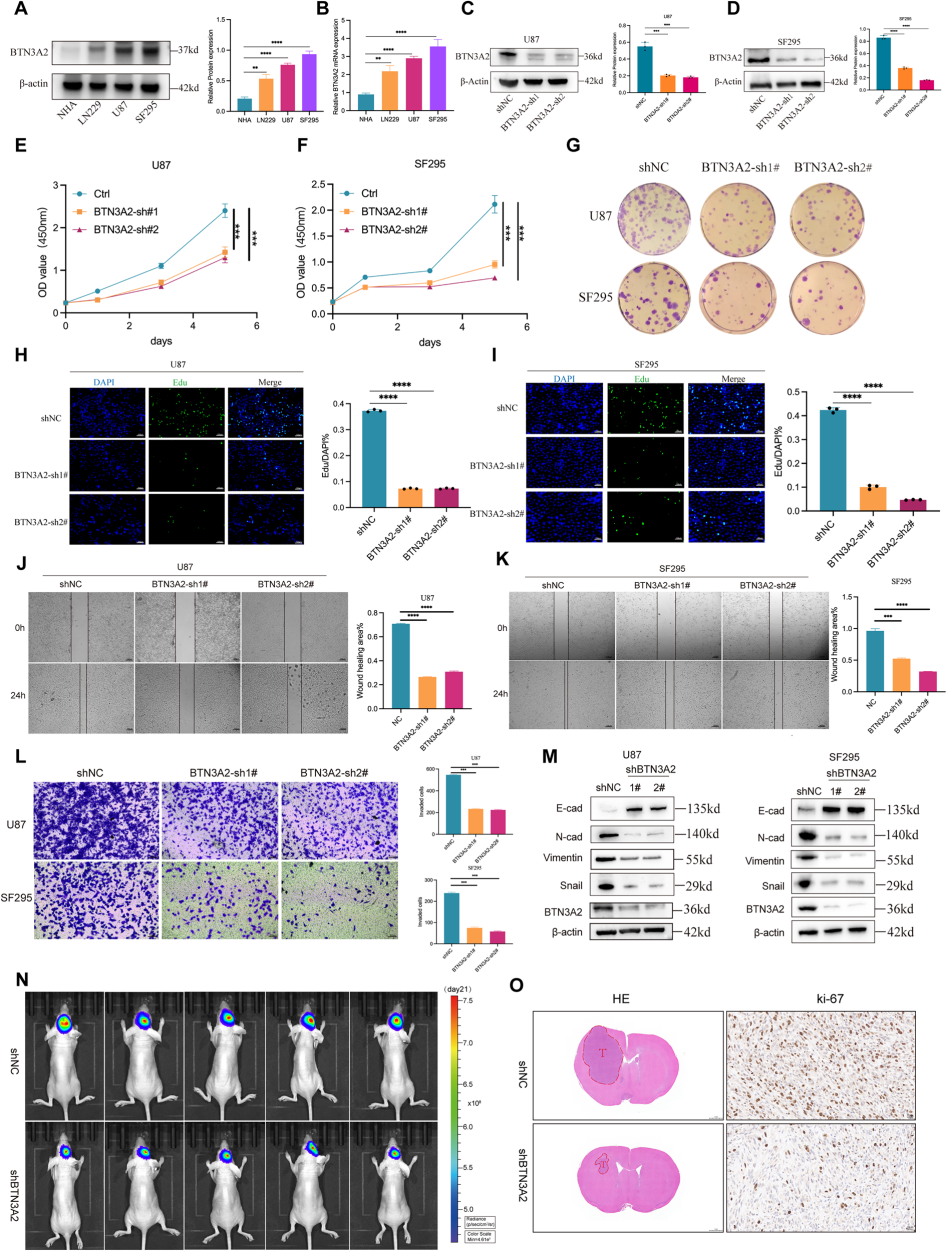
BTN3A2 promots glioma cell growth in vitro and in vivo. **A,B** The protein and mRNA expression of BTN3A2 in NHA cell and human glioma cells (LN229,U87 and SF295). **C**, **D** BTN3A2 knockdown in U87 and SF295 cells was confirmed by Western blot. **E**, **F** CCK-8 assays were performed to determine the effects of BTN3A2 knockdown on the proliferation of U87 and SF295 cells. **G** BTN3A2 knockdown inhibits the colony formation ability of U87 and SF295 in vitro. **H**, **I** BTN3A2 knockdown reduces DNA synthesis in U87 and SF295 cells.EdU staining (green) marks proliferating cells; Nuclei are counterstained with Hoechst 33342 (blue). Scale bar: 100 μm. Quantification of the EdU-positive cells is shown in the right panel. **J–L** Wound healing and Transwell assays of cells with BTN3A2 knockdown. Scale bars: 100 μm. **M** Expression levels of EMT-related proteins (Snail1, Vimentin, N-cadherin, E-cadherin) in BTN3A2 knockdown U87 and SF295 cells. **N** Representative bioluminescence images of orthotopic intracranial xenografts. **O** Representative images of H&E staining and Ki-67 immunohistochemical (IHC) staining. Scale bars,1 mm (H&E) and 20 µm (IHC). All images represented as the mean ± SD of three independent experiments; *P < 0.05; **,*P* < 0.01; ***P < 0.001; ****P < 0.0001.

Current research on BTN3A2 emphasizes its role in phosphoantigen-mediated γδT cell activation, although its non-immune-related roles in glioma remain unreported. This investigation identified the expression of BTN3A2 in glioma cell lines (LN229, U87, SF295) and normal human astrocytes (NHA) using Western blotting and RT-PCR, revealing that BTN3A2 expression in glioma cells was markedly elevated compared to normal cells (Fig. 2A, B). Subsequently, we employed shRNA to create U87 and SF295 glioma cell lines with a stable knockdown of BTN3A2 (Fig. 2C, D).

Furthermore, Immunofluorescence labeling revealed that BTN3A2 was mostly located in the cytoplasm of glioma cells (Fig. S2A). CCK8 assays demonstrated that the suppression of BTN3A2 inhibited cell growth (Fig. 2E, F). Subsequently, we conducted a cloning assay, and the findings indicated that BTN3A2 knockdown impeded cell cloning (Fig. 2G). EdU staining indicated that the DNA replication rate in BTN3A2 knockdown cells was markedly diminished (Fig. 2H, I). Wound healing assays and Transwell migration assays demonstrated that the knockdown of BTM3A2 impeded the migration and invasion of U87 and SF295 cells (Fig. 2J–L). Western blotting revealed decreased expression of vimentin, N-cadherin, and snail, alongside increased E-cadherin (Fig. 2M). The flow cytometry apoptosis experiment subsequently revealed that the suppression of BTN3A2 markedly enhanced the apoptosis of glioma cells (Figure S2B-D). Cell cycle analysis revealed that the knockdown of BTN3A2 resulted in a pronounced G2/M phase arrest in SF295 glioma cells (Fig. S2E, F). The Western blot results indicated a general decline in the expression levels of the regulatory elements P21 and P27, which are intimately associated with G2/M cell cycle arrest (Fig. S2G–J).

In vivo experiments consistently demonstrated that BTN3A2 plays a critical role in promoting glioma growth. Specifically, in an orthotopic intracranial xenograft model, BTN3A2 knockdown suppressed tumor progression compared with the NC group, as indicated by reduced intracranial tumor burden assessed by bioluminescence imaging (Fig. 2N). Furthermore, histopathological analysis revealed that tumors from shBTN3A2 cells had smaller areas and less aggressive growth, as assessed by H&E staining, and showed a significant reduction in Ki-67 expression, indicating impaired proliferative activity in vivo (Fig. 2O). Consistent with the intracranial model findings, a subcutaneous xenograft model showed that BTN3A2 knockdown led to significantly reduced tumor growth and lower tumor weights compared with controls (Fig. S2K–M). Additionally, immunohistochemical analyses of subcutaneous tumors demonstrated reduced Ki-67 staining together with increased cleaved-caspase-3 expression, indicating enhanced apoptosis following BTN3A2 knockdown (Fig. S2N). Collectively, these findings from both orthotopic and subcutaneous models confirm the pro-tumorigenic role of BTN3A2 in glioma growth and proliferation in vivo.

### Hypoxia directly induces BTN3A2 expression via HIF-1α

**Fig. 3:**
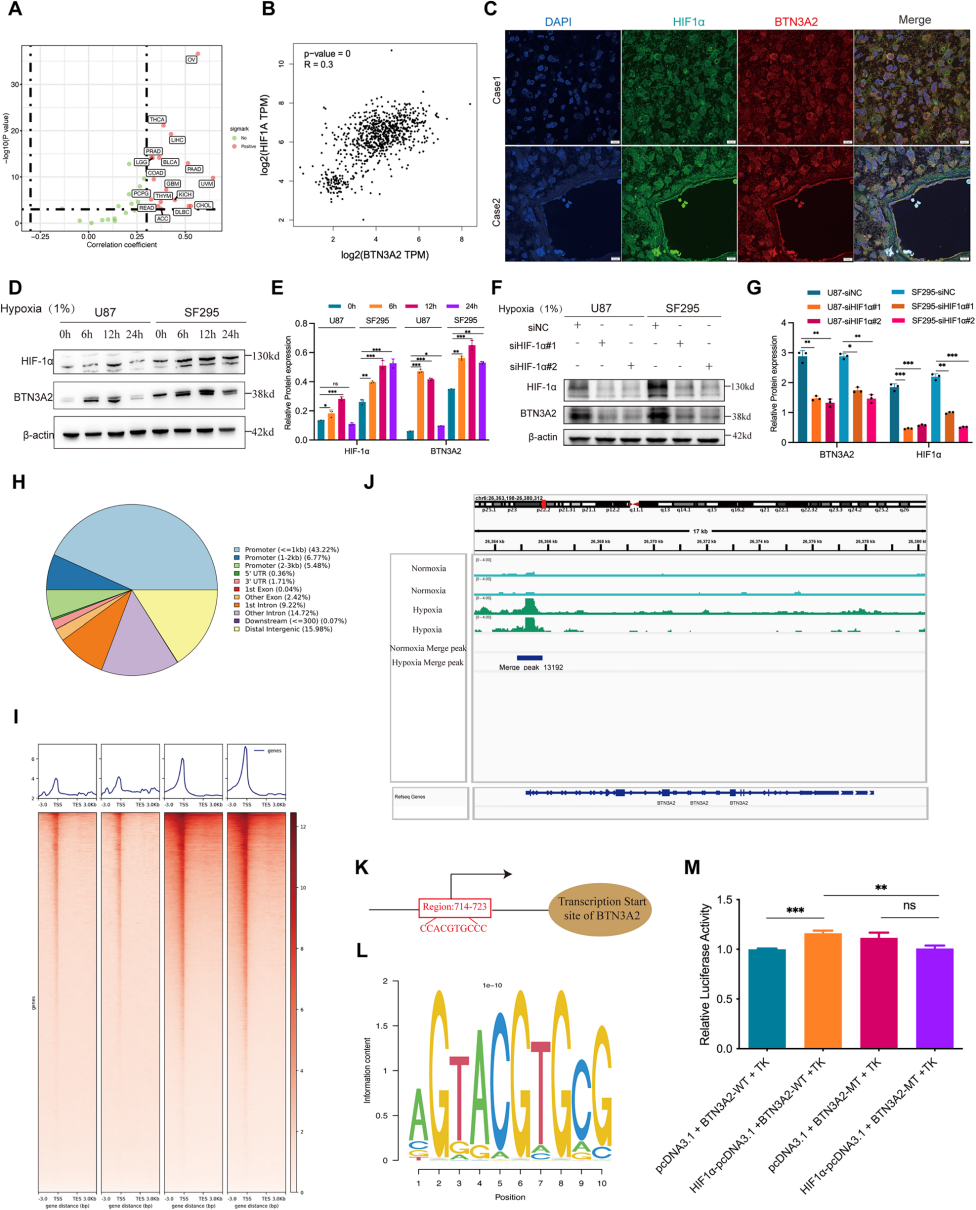
Hypoxia induced BTN3A2 in a HIF-1α dependent manner. **A**, **B** Scatter plots depicting the expression correlation of BTN3A2 with HIF-1α signature scores. **C** Coimmunofluorescence staining of HIF-1α(green) and BTN3A2(red) in human Glioma tissue.Scale bars,10 μm.**D**, **E** Western blot analysis of HIF-1α and BTN3A2 protein expression dynamics in U87 and SF295 cells exposed to hypoxia for (0, 6, 12, 24 h). **F**, **G** Western blot analysis of HIF-1α and BTN3A2 protein levels following HIF-1α knockdown with specific siRNA in U87 and SF295 cells. **H–J** Cut&Tag analysis of HIF‑1α binding sites showing genome-wide distribution under normoxia and hypoxia, with prominent enrichment at the BTN3A2 promoter region. Aggregation plots and heatmaps illustrate increased HIF‑1α Cut&tag signal intensity around the transcription start site (TSS) and transcription end site (TES) of BTN3A2 under hypoxic conditions. **K**, **L** Hypoxia response elements (HREs) on the region of BTN3A2 promoter and the motif result. **M** Luciferase reporter assays using the BTN3A2 promoter in U87 cells.All image represented as the mean ± SD of three independent experiments; *P < 0.05; *P < 0.01; ***P < 0.001; ****P < 0.0001, ns, no significance.

Hypoxia is prevalent in glioma tissues and significantly contributes to the growth and invasion of gliomas [23]. To investigate the correlation between BTN3A2 expression and hypoxia, we initially conducted standardized RNA-seq analysis using the TCGA and CGGA databases. The findings indicated a substantial correlation between BTN3A2 expression and many hypoxia-related indicators, including HIF-1α, VEGFA, CA9, and PGK1 (Fig. 3A, B; Fig. S3A–D). Additionally, KEGG pathway enrichment analysis of RNA-seq data following BTN3A2 knockdown indicated significant downregulation of the HIF-1α signaling pathway, suggesting that BTN3A2 may participate in hypoxia-related regulatory networks (Fig.S3E, F). Immunofluorescence staining of glioma patient tissue samples further revealed prominent co-localization of BTN3A2 and HIF-1α within cells (Fig. 3C), supporting a direct association between these molecules in transcriptional regulation.

To further assess whether BTN3A2 is a direct target gene of HIF-1α, U87 and SF295 cells were cultured in a hypoxic environment containing 1% O_2_. Under hypoxic conditions, BTN3A2 expression in both cell lines increased in a time-dependent manner. Subsequently,we transfected cells with two independent HIF-1α-specific siRNAs (si-HIF-1α#1 and #2). Western blot analysis confirmed that both siRNAs significantly attenuated the hypoxia-induced upregulation of BTN3A2 (Fig. 3D–G). To investigate whether BTN3A2 is a direct transcriptional target of HIF-1α under hypoxic conditions, CUT&Tag sequencing was performed. Hypoxia treatment induced a prominent enrichment peak in the BTN3A2 promoter (Fig. 3H–I), with intensity changes depicted in Fig. S3F. A detailed analysis of the promoter differential peaks revealed the hypoxia response element of HIF-1α inside the 714–723 bp region of the BTN3A2 gene promoter (Figure. J-L). Furthermore, we developed wild-type (WT) and mutant (Mut) promoter reporter gene vectors (HIF-1α-Luc) utilizing the promoter sequence that encompasses hypoxia response elements (HREs). The dual luciferase test results indicated that in U87 cells, transfection with the mutant vector markedly reduced the hypoxia-induced BTN3A2 promoter activity in comparison to cells transfected with the wild-type vector (Fig. 3M). Collectively, these findings indicate that BTN3A2 is a direct transcriptional target of HIF-1α.

### BTN3A2 knockdown increases the sensitivity of glioma cells to chemotherapy both in vitro and in vivo

**Fig. 4:**
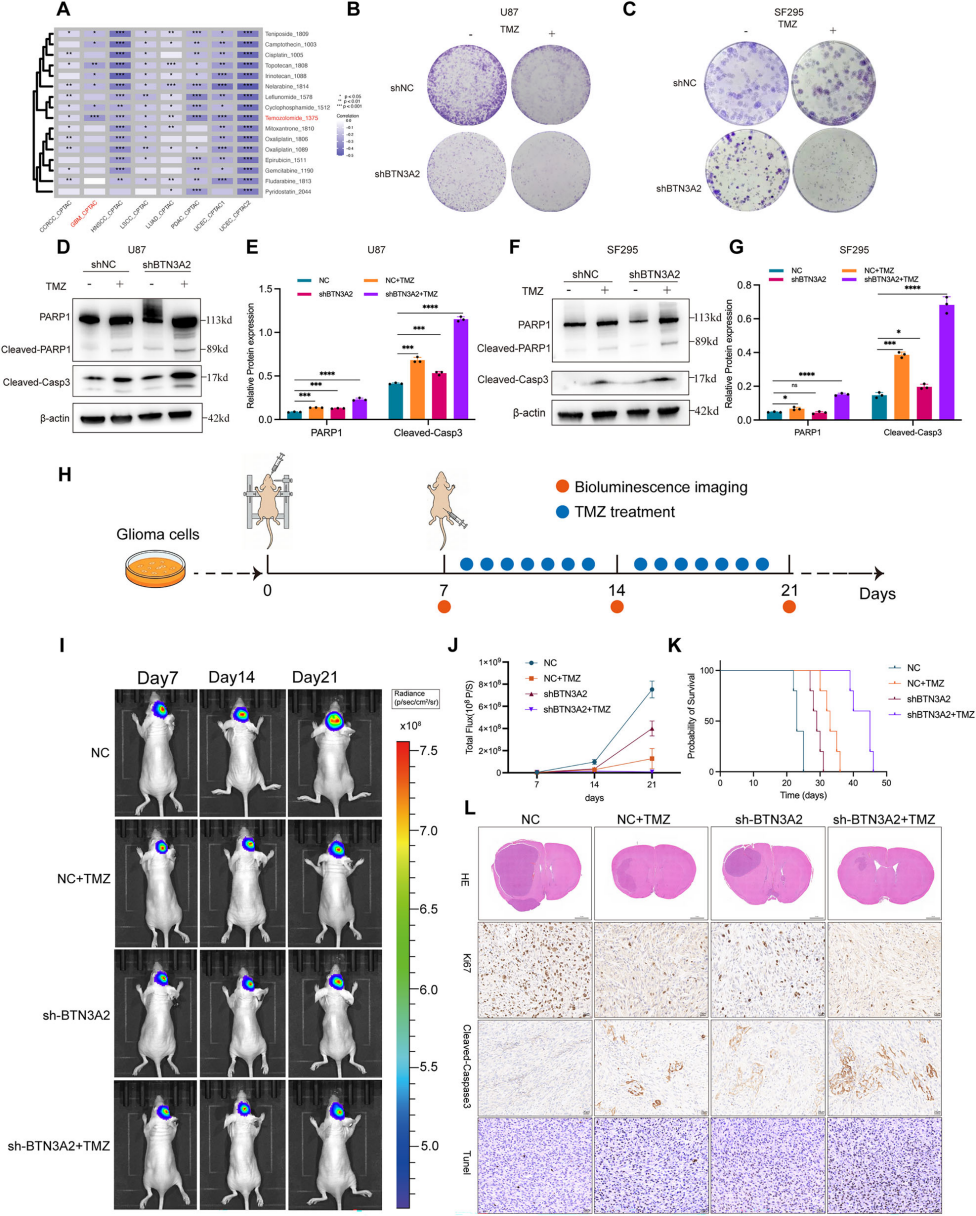
BTN3A2 knockdown sensitizes glioma cells to Temozolomide-induced apoptosis in vitro and vivo. **A** Analysis of BTN3A2 associated drug sensitivity in glioma using the ProteoCancer Analysis Suite (PCAS). **B, C** Knockdown of BTN3A2 enhances TMZ induced suppression of colony formation in glioma cells. **D–G** BTN3A2 knockdown combined with TMZ promotes apoptosis in glioma cells. **H** Schematic illustration of the experimental design and treatment schedule for the orthotopic intracranial xenograft model. **I** Representative longitudinal bioluminescence images of orthotopic gliomas from each treatment group at the indicated time points. **J** Quantification of bioluminescence signal intensity showing intracranial tumor growth dynamics. **K** Kaplan–Meier survival analysis of mice bearing orthotopic gliomas. **L** Representative H&E staining and immunohistochemical analysis of Ki-67, cleaved caspase-3 and TUNEL assay in orthotopic tumor tissues. Scale bar:1 mm (H&E) and 20 µm (IHC). All image represented as the mean ± SD of three independent experiments; *P < 0.05; **,*P* < 0.01; ***P < 0.001; ****P < 0.0001；ns no significance.

To evaluate the effect of BTN3A2 on glioma chemosensitivity, the ProteoCancer Analysis Suite (PCAS) database (https://jingle.shinyapps.io/PCAS/) [24] was initially utilized to systematically assess the drug sensitivity of BTN3A2 across multiple cancer types. The analysis revealed that TMZ exhibited the greatest chemotherapeutic sensitivity in glioblastoma (Fig. 4A). Clonal formation experiments subsequently demonstrated that the clonal formation capacity of U87 and SF295 cells infected with lentivirus carrying shRNA targeting the BTN3A2 gene was markedly diminished following combined TMZ treatment, indicating that BTN3A2 knockdown significantly enhanced the suppression of TMZ-induced U87 and SF295 cell clones (Fig. 4B, C). Subsequent Western blot analyses demonstrated that the reduction of BTN3A2, in conjunction with TMZ, markedly enhanced TMZ-induced apoptosis in glioma cells, along with an elevation in apoptosis-related markers, including cleaved caspase 3 and cleaved PARP (Fig. 4D–G). The observations suggest that the suppression of BTN3A2 can synergize with TMZ to improve chemotherapeutic efficacy by activating apoptotic pathways.

To further investigate whether BTN3A2 depletion enhances the therapeutic efficacy of TMZ in vivo, an orthotopic intracranial xenograft model was established, and the experimental timeline is schematically illustrated in Fig. 4H. Tumor progression was monitored longitudinally by bioluminescence imaging at the indicated time points. Representative images showed that TMZ treatment reduced intracranial tumor burden, and this effect was markedly greater in mice bearing shBTN3A2 tumors than in control groups (Fig. 4I).

Quantitative analysis of bioluminescence signal intensity confirmed that combined BTN3A2 knockdown and TMZ treatment significantly suppressed tumor growth over time compared to either intervention alone (Fig. 4J). Kaplan–Meier survival analysis showed that TMZ treatment significantly prolonged survival in tumor-bearing mice. The greatest survival benefit was observed in the shBTN3A2 + TMZ group (Fig. 4K). This finding indicates that BTN3A2 depletion sensitizes glioma cells to TMZ therapy in the intracranial setting.

Histopathological and immunohistochemical analyses of brain tumor sections showed that tumors from the shBTN3A2 + TMZ group had fewer tumor cells and less invasive growth, as determined by H&E staining. Additionally, there was a marked decrease in proliferating (Ki-67–positive) cells and a notable increase in both apoptosis (cleaved-caspase 3 staining) and TUNEL assasy (Fig. 4L). Together, these results indicate that shBTN3A2+TMZ treatment effectively reduced tumor growth and increased cell death in vivo. Consistent results were also obtained in a subcutaneous xenograft model treated with TMZ (Supplementary Fig. S4), supporting BTN3A2’s role in TMZ resistance in glioma.

### Inhibition of BTN3A2 enhances TMZ-mediated DNA damage in glioma cells

**Fig. 5:**
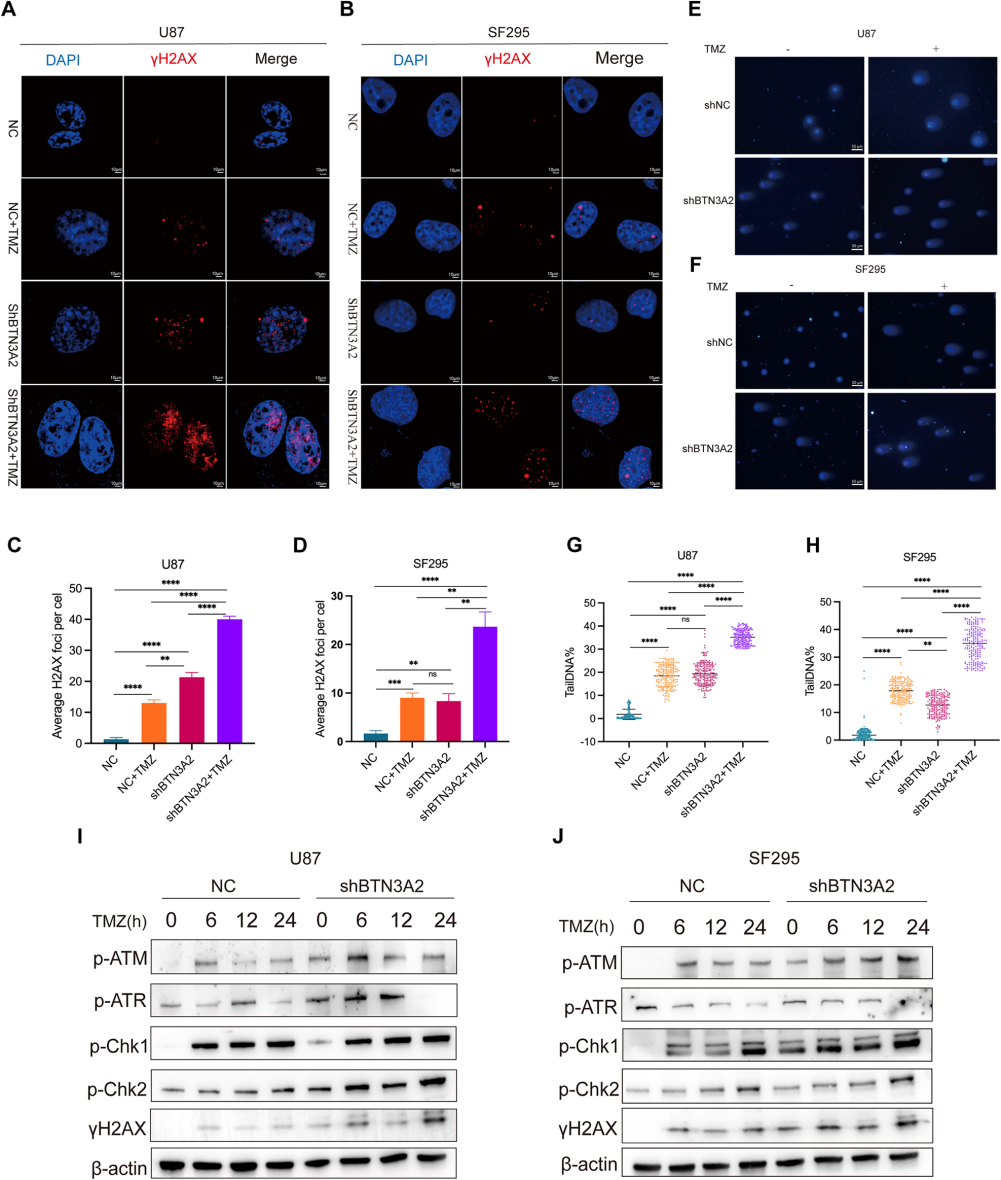
BTN3A2 knockdown aggravates TMZ-induced DNA damage and enhances activation of DNA damage response pathways in glioma cells. **A–D** Immunofluorescence staining of γ-H2AX foci in U87 and SF295 cells with or without BTN3A2 knockdown after TMZ treatment. Quantification of γ-H2AX foci per nucleus is shown below. Knockdown of BTN3A2 significantly increased the number of γ-H2AX foci, indicating enhanced DNA double-strand breaks. Scale bars:10 μm. **E–H** Comet assay showing that BTN3A2 knockdown significantly prolonged the tail length of comet structures in U87 and SF295 cells following TMZ treatment, suggesting increased DNA damage. Quantitative analysis of tail moment is shown below. **I**, **J** Western blot analysis of key DDR pathway proteins in BTN3A2 knockdown and control cells at different time points after TMZ treatment. Statistical analyses of protein levels are presented in Supplementary Fig. S4C, D. All image represented as the mean ± SD of three independent experiments; *P < 0.05; **P < 0.01; ***P < 0.001; ****P < 0.0001； ns no significance.

Considering that TMZ primarily exerts its chemotherapeutic impact through the induction of DNA damage [25], we hypothesized that BTN3A2 may influence cellular apoptosis and chemotherapy susceptibility by modulating DNA damage-associated signaling pathways. To validate this hypothesis, we compared the differentially expressed genes following BTN3A2 knockdown with the established DNA damage response (DDR) gene set derived from sequencing results, revealing a significant correlation between BTN3A2 knockdown and alterations in the expression of several critical DDR markers, including CDK2, RAD51, CHEK1, and CHAF1B. (Fig. S5A, B). Consistent with expectations, IF labeling revealed a large increase in the frequency of γ-H2AX foci in BTN3A2 knockdown cells (Fig. 5A–D). Likewise, the reduction of BTN3A2 in U87 and SF295 cells markedly extended the length of the comet tail caused by TMZ treatment, thereby further substantiating the exacerbation of DNA damage (Fig. 5E–H). Furthermore, we conducted a systematic analysis of the dynamic alterations in DDR signaling pathway proteins following DNA damage. Activated ATM and ATR kinases phosphorylate H2AX, resulting in the formation of γH2AX, which enhances DNA damage signals. In U87 and SF295 glioma cells with BTN3A2 knockdown, the phosphorylation levels of ATM, ATR, CHK1, CHK2, and H2AX (p-ATM, p-ATR, p-CHK1, p-CHK2, γH2AX, respectively) were elevated following TMZ treatment and persisted for a minimum of 24 hours (Fig. 5I-J). The statistical analysis of protein levels is presented in (Fig. S5C, D). The aforementioned data suggest that the inhibition of BTN3A2 can augment the induction of DNA damage response by TMZ.

### BTN3A2 modulates DNA damage in glioma cells via the p-AKT/SP1/RAD51 axis

**Fig. 6:**
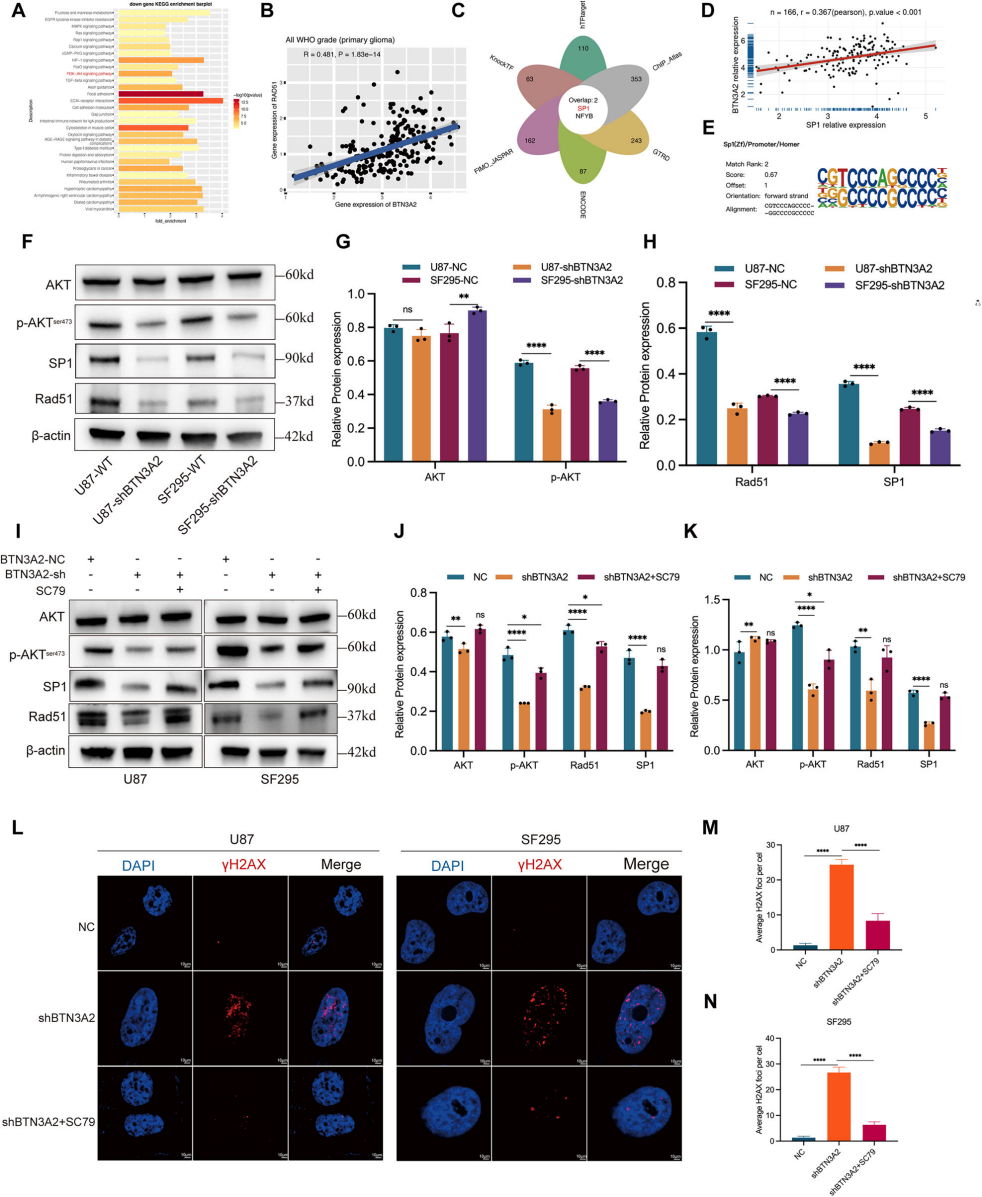
BTN3A2 regulates DNA damage response through the p-AKT/SP1/RAD51 signaling axis in glioma cells. **A** KEGG pathway enrichment analysis showing that genes differentially expressed after BTN3A2 knockdown were mainly enriched in the PI3K/AKT signaling pathway. **B–E** Motif analysis based on sequencing data revealing a significant reduction in the frequency of SP1 binding motifs in BTN3A2 knockdown samples, suggesting a regulatory relationship between BTN3A2 and SP1. **F–H** Western blot analysis demonstrating that BTN3A2 knockdown markedly reduced the protein level of SP1 and specifically downregulated the phosphorylation of AKT at Ser473, while total AKT levels remained unchanged. Representative blots and quantitative analyses are shown o the right. **I–K** Rescue experiments with the PI3K/AKT pathway agonist SC79 in BTN3A2 knockdown U87 and SF295 cells. SC79 treatment restored p-AKT levels and reversed the downregulation of SP1 and RAD51 protein expression induced by BTN3A2 knockdown. **L–N** Immunofluorescence staining of γ-H2AX foci in glioma cells. Quantification of γ-H2AX foci per nucleus is shown on the right.All image represented as the mean ± SD of three independent experiments; *P < 0.05; **P < 0.01; ***P < 0.001; ****P < 0.0001；ns no significance.

KEGG pathway enrichment analysis indicated that genes differentially expressed following BTN3A2 knockdown were predominantly enriched in the PI3K/AKT signaling pathway (Fig. 6A), suggesting that BTN3A2 may exert regulatory effects via this pathway. The transcription factor SP1 is known to positively regulate RAD51 expression, while activation of the PI3K/AKT pathway enhances SP1 stability, promoting its nuclear accumulation and transcriptional activity [19, 20]. Sequencing data from this study demonstrated a positive correlation between BTN3A2 and SP1 expression levels, implying a potential role for BTN3A2 in SP1 regulation. Following the knockdown of BTN3A2, the prevalence of the SP1 binding motif in the samples was markedly diminished (Fig. 6B–E). To validate the aforementioned hypothesis, we performed tests on U87 and SF295 glioma cell lines and discovered that the downregulation of BTN3A2 markedly reduced the expression level of SP1 protein. Simultaneously, we assessed the expression and phosphorylation state of AKT, revealing that BTN3A2 knockdown particularly decreased the level of p-AKT (Ser473), but the overall AKT protein level remained relatively unchanged (Fig. 6F–H).

**Fig. 7:**
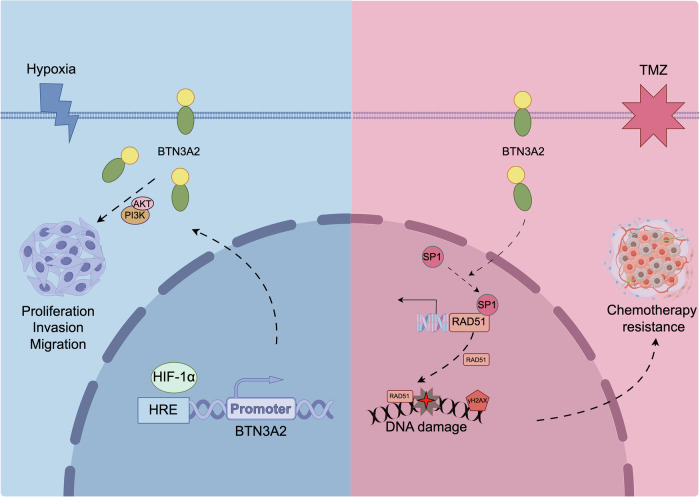
Schematic mechanism overview of hypoxia-induced BTN3A2 expression and its independent role in glioma chemoresistance via the p-AKT/SP1/RAD51 pathway.

To further elucidate the involvement of p-AKT in the regulation of SP1/RAD51 by BTN3A2, rescue experiments were performed using the PI3K/AKT pathway agonist SC79. The findings indicated that SC79 administration successfully reinstated p-AKT expression in BTN3A2 knockdown cells (BTN3A2-sh) while concurrently reversing the downregulation of SP1 and RAD51 protein levels (Fig. 6I–K). Furthermore, immunofluorescence detection revealed that following SC79 activation of p-AKT, the development of foci for the DNA damage marker γH2AX in BTN3A2-sh cells was markedly diminished in comparison to the control group (Fig. 6L–N). In conclusion, our findings collectively indicate that BTN3A2 modulates the DNA damage response in glioma cells via increasing the p-AKT/SP1/RAD51 signaling pathway (Fig. 7).

## Discussion

In this study, we identify BTN3A2 as a previously unrecognized cell-intrinsic regulator of glioma progression and therapeutic resistance. Through an integrated analysis combining clinical correlation, mechanistic investigation, and orthotopic in vivo validation, we demonstrate that BTN3A2 is upregulated in high-grade gliomas and functions as a key effector of hypoxia adaptation. Importantly, our findings extend the biological significance of BTN3A2 beyond its established immunomodulatory roles and delineate a hypoxia-responsive signaling axis linking HIF-1α activation to enhanced DNA damage response capacity. Together, these results position BTN3A2 as a molecular mediator that connects microenvironmental stress to treatment resistance in glioma.

The BTN3A family has been predominantly studied in the context of tumor immunology, particularly for its role in regulating Vγ9Vδ2 T cell activation [26, 27]. Accordingly, therapeutic strategies targeting BTN3A have largely focused on immune engagement. However, accumulating evidence suggests that molecules traditionally classified as immune checkpoints may also exert tumor-intrinsic functions independent of immune surveillance [28–30]. Our data support this emerging paradigm in glioma, demonstrating that BTN3A2 promotes tumor cell proliferation and invasion in immunodeficient experimental systems. This distinction is especially relevant in glioblastoma, where the immunologically “cold” microenvironment often limits the efficacy of immunotherapy, underscoring the importance of cell-intrinsic survival mechanisms in driving disease progression and therapeutic resistance.

Hypoxia is a defining feature of the glioma microenvironment and a well-established driver of tumor aggressiveness and chemoresistance [31]. In this study, we identify BTN3A2 as a direct transcriptional target of HIF-1α, thereby positioning it downstream of hypoxia signaling. This finding provides mechanistic insight into how oxygen deprivation is translated into adaptive cellular programs. Rather than serving solely as a passive hypoxia-associated marker, BTN3A2 appears to function as an active participant that reinforces glioma cell survival under hypoxic stress conditions.

A central finding of this study is the mechanistic link between BTN3A2 and temozolomide resistance through the regulation of DNA repair pathways. TMZ-induced cytotoxicity primarily depends on the accumulation of DNA double-strand breaks, which are resolved by homologous recombination in proliferating cells [32]. We demonstrate that BTN3A2 upregulation is associated with increased expression of RAD51, a key recombinase in the homologous recombination machinery, thereby facilitating efficient repair of TMZ-induced genotoxic lesions. Furthermore, our data indicate that this process is mediated, at least in part, through the p-AKT/SP1 signaling axis, linking BTN3A2 activity to transcriptional control of DNA repair capacity.

Although the precise molecular interface between BTN3A2 and AKT activation requires further clarification, it is plausible that intracellular domains of BTN3A2 contribute to the assembly or stabilization of signaling complexes that promote AKT phosphorylation and downstream SP1 activation. Previous structural and biochemical studies suggest that the B30.2 domain can function as a protein–protein interaction module, supporting the possibility that BTN3A2 indirectly facilitates AKT signaling. Further structural and biochemical analyses will be necessary to define these interactions at higher resolution.

Importantly, our findings were validated in an orthotopic intracranial xenograft model established in BALB/c nude mice. Unlike subcutaneous implantation, orthotopic models preserve critical features of the brain microenvironment, including blood–brain barrier constraints and tissue-specific stromal interactions [33]. The consistent tumor-suppressive effect observed upon BTN3A2 silencing in this context supports the robustness of its tumor-intrinsic function in glioma progression and therapeutic resistance.

From a clinical perspective, the identification of BTN3A2 as an independent prognostic factor suggests its potential utility in risk stratification for glioma patients receiving TMZ-based therapy. Therapeutically, targeting BTN3A2-associated signaling may represent a strategy to attenuate adaptive DNA repair responses and improve treatment efficacy. Rather than directly targeting proliferative pathways, such an approach could disrupt stress-adaptive mechanisms that enable tumor persistence under therapeutic pressure.

Several limitations of this study should be acknowledged. Although the orthotopic model more accurately reflects the glioma microenvironment, the use of BALB/c nude mice precludes assessment of BTN3A2-mediated immune regulation. Given that BTN3A2 may exert dual roles by influencing both tumor-intrinsic survival and anti-tumor immunity, future studies employing immunocompetent or humanized models will be required to define the net therapeutic impact of BTN3A2 targeting. In addition, the development of strategies capable of selectively modulating the non-immune functions of BTN3A2 warrants further investigation.

## Conclusion

This study reveals BTN3A2 as a novel cell-intrinsic regulator of glioma growth and treatment resistance. Our findings demonstrate that BTN3A2 is upregulated in high-grade gliomas and promotes tumor cell survival under hypoxic and genotoxic stress conditions. Mechanistically, BTN3A2 acts downstream of HIF-1α to enhance DNA damage response capacity, thereby shifting the balance away from cell death toward survival. Validation in an orthotopic intracranial glioma model further underscores the physiological relevance of these observations. Collectively, this work reveals a novel hypoxia-adaptive survival pathway in glioma and highlights BTN3A2 as a potential biomarker and therapeutic vulnerability associated with treatment resistance.

## Supplementary information

Figure legends

Uncropped blots

FigureS1

FigureS2

FigureS3

FigureS4

FigureS5

TableS1

Table S2

## Data Availability

The datasets used and/or analysed during the current study are available from the corresponding author on reasonable request.
