# Supplementary material for: Hypoxia-induced BTN3A2 promotes glioma progression and chemoresistance via AKT/SP1/RAD51-mediated DNA damage
Source: Cell Death Dis. 2026 Apr 11;17(1):469. doi: 10.1038/s41419-026-08729-7 (PMC13181034; doi:10.1038/s41419-026-08729-7)
Supplement: Supplementary file 1 — Figure legends [file 41419_2026_8729_MOESM1_ESM.docx]

**Supplemental Figure 1:**

Prognostic significance and expression patterns of BTN3A2 in glioma patients across multiple datasets.**A-C** BTN3A2 mRNA expression levels in gliomas of different histological subtypes and grades based on CGGA datasets.**D-E** Univariate and multivariate Cox regression analyses showing that BTN3A2 expression is an independent prognostic factor for overall survival in glioma patients.**F-H** Kaplan–Meier survival curves demonstrating that high BTN3A2 expression is significantly associated with worse overall survival in glioma patients from TCGA, CGGA, and REMBRANDT cohorts. Statistical significance was determined using log-rank test for Kaplan–Meier analysis and Cox proportional hazards regression.

**Supplemental Figure2:**

BTN3A2 knockdown promotes apoptosis and induces G2/M phase arrest in glioma cells.**A** Immunofluorescence staining showing the cytoplasmic localization of BTN3A2 in U87 glioma cells. **B-D** Flow cytometry analysis indicating increased apoptosis rates in BTN3A2 knockdown U87 and SF295 cells compared with control cells. **E-F** Cell cycle distribution analysis demonstrating that BTN3A2 knockdown induces significant G2/M phase arrest in SF295 glioma cells. **G-J** Western blot analysis showing that knockdown of BTN3A2 leads to decreased expression levels of cell cycle regulators P21 and P27.**K** Images of the xenograft tumors formed in nude mice injected with BTN3A2-shRNA cells and control cells.**L-M** Tumor volume and tumor weight were calculated. **N** Representative images of IHC staining of ki-67,Cleaved-Casp3 and P-Akt. Scale bars,20 μm. All images represented as the mean ± SD of three independent experiments;Statistical analysis was performed using Student’s t-test.*,*P* < 0.05;**,*P* < 0.01;***,*P* < 0.001;****,*P* < 0.0001

**Supplemental Figure3:**

BTN3A2 expression is positively associated with hypoxia-related markers and the HIF-1α signaling pathway in gliomas.**A-D** Correlation analysis showing significant positive associations between BTN3A2 mRNA expression and hypoxia-related markers (HIF-1α, VEGFA, CA9, and PGK1) in CGGA datasets. **E-F** KEGG pathway enrichment analysis of RNA-seq data following BTN3A2 knockdown revealing significant downregulation of the HIF-1α signaling pathway and CUT&Tag peak relative to the transcription start site.Correlation coefficients were calculated using Spearman’s correlation test.*,*P* < 0.05;**,*P* < 0.01;***,*P* < 0.001;****,*P* < 0.0001

**Supplemental Figure4:**

BTN3A2 depletion enhances temozolomide sensitivity in a subcutaneous xenograft model.**A** Schematic of the in vivo experimental design.**B-C** Representative images of tumors from each treatment group.**D** Final tumor weights.**E** Tumor growth inhibition analysis.**F** Immunohistochemical staining of cleaved caspase-3, TUNEL assay, and Ki-67 in xenograft tumor tissues.Scale bar: 25 µm. All image represented as the mean ± SD of three independent experiments; **P* < 0.05;**,*P* < 0.01;***,*P* < 0.001;****,*P* < 0.0001；ns, no significance

**Supplemental Figure5:**

BTN3A2 knockdown modulates the DNA Damage Response pathway.**A** Venn diagram depicting the intersection between consistently downregulated genes in BTN3A2-knockdown cells and DNA damage response (DDR) genes (51 genes).**B** Correlation analysis showing significant positive associations between BTN3A2 mRNA expression and DDR markers (RAD51, CHEK1, CDK2, and CHAF1B) in CGGA datasets.**C-D** Densitometric quantification of DDR protein phosphorylation dynamics in BTN3A2-knockdown versus control glioma cells after Temozolomide treatment.Correlation coefficients were calculated using Spearman’s correlation test.
