# Supplementary material for: Hypoxia-induced BTN3A2 promotes glioma progression and chemoresistance via AKT/SP1/RAD51-mediated DNA damage
Source: Cell Death Dis. 2026 Apr 11;17(1):469. doi: 10.1038/s41419-026-08729-7 (PMC13181034; doi:10.1038/s41419-026-08729-7)
Supplement: Supplementary file 2 — Uncropped blots [file 41419_2026_8729_MOESM2_ESM.docx]

**Table S2 Details of primary antibodies**

| Primary antibody | Application | Dilution Ratio | Lot number | Company |
| --- | --- | --- | --- | --- |
| BTN3A2 | WB / IHC | WB 1:1000;  IHC 1:100 | ab118051 | Abcam |
| β-actin | WB | 1：1000 | [20536-1-AP](http://www.ptgcn.com/products/ACTB-Antibody-20536-1-AP.htm) | Proteintech |
| Vimentin | WB | 1：1000 | 10366-1-AP | Proteintech |
| Snail1 | WB | 1：1000 | #3879 | Cell Signaling Technology |
| E-cadherin | WB | 1：1000 | # 3195 | Cell Signaling Technology |
| N-cadherin | WB | 1：1000 | 22018-1-AP | Proteintech |
| HIF-1α | WB/CUT&Tag/  IF/IHC | WB 1:1000; CUT&Tag 1:50  IF/IHC: 1:100 | #36169S | Cell Signaling Technology |
| IgG | CUT&Tag | 1：100 | AF1935 | R&D Systems |
| Caspase 3 | WB | 1：1000 | 19677-1-AP | Proteintech |
| Cleaved-caspase3 | WB/IHC | WB 1:1000;  IHC 1:100 | ab32042 | Abcam |
| p21 | WB | 1：1000 | 10355-1-AP | Proteintech |
| P27 | WB | 1：1000 | 25614-1-AP | Proteintech |
| CDC25C | WB | 1：1000 | 16485-1-AP | Proteintech |
| P-CDC25C | WB | 1：1000 | #4901 | Cell Signaling Technology |
| PARP | WB | 1：1000 | 13371-1-AP | Proteintech |
| ki-67 | IHC | 1：100 | 27309-1-AP | Proteintech |
| p-ATM | WB | 1：1000 | # 5883 | Cell Signaling Technology |
| p-ATR | WB | 1：1000 | # 30632 | Cell Signaling Technology |
| γH2AX | WB/IHC/IF | WB 1:1000;  IHC/IF 1:100 | # 9718 | Cell Signaling Technology |
| p-CHK1 | WB | 1：1000 | # 12302 | Cell Signaling Technology |
| p-CHK2 | WB | 1：1000 | # 2661 | Cell Signaling Technology |
| Rad51 | WB | 1：1000 | 14961-1-AP | Proteintech |
| SP1 | WB | 1：1000 | # 9389 | Cell Signaling Technology |
| AKT | WB | 1：1000 | #9272S | Cell Signaling Technology |
| p-AKT(ser473) | WB | 1：1000 | #9271S | Cell Signaling Technology |
