# Supplementary material for: Hypoxia-induced BTN3A2 promotes glioma progression and chemoresistance via AKT/SP1/RAD51-mediated DNA damage
Source: Cell Death Dis. 2026 Apr 11;17(1):469. doi: 10.1038/s41419-026-08729-7 (PMC13181034; doi:10.1038/s41419-026-08729-7)

Uncropped blots related to Figure2

Fig.2A

BTN3A2

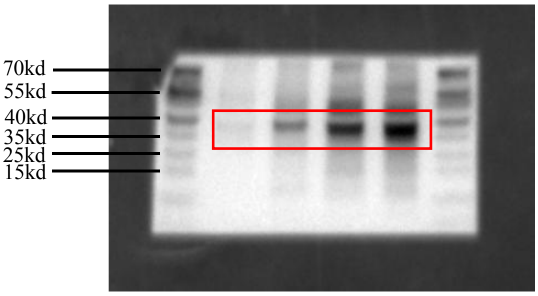

ACTIN

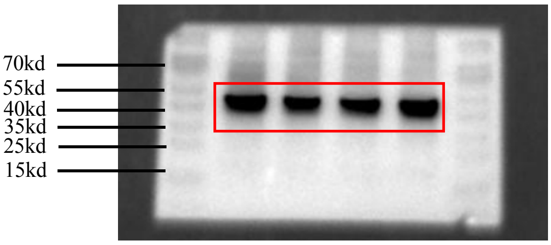

Fig.2C

BTN3A2

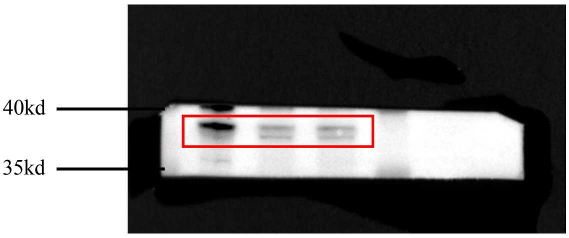

ACTIN

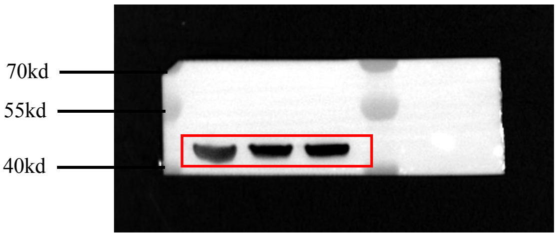

Fig.2D

BTN3A2

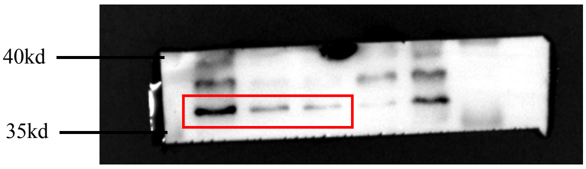

ACTIN

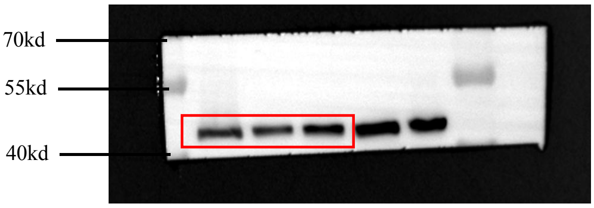

Fig.2M

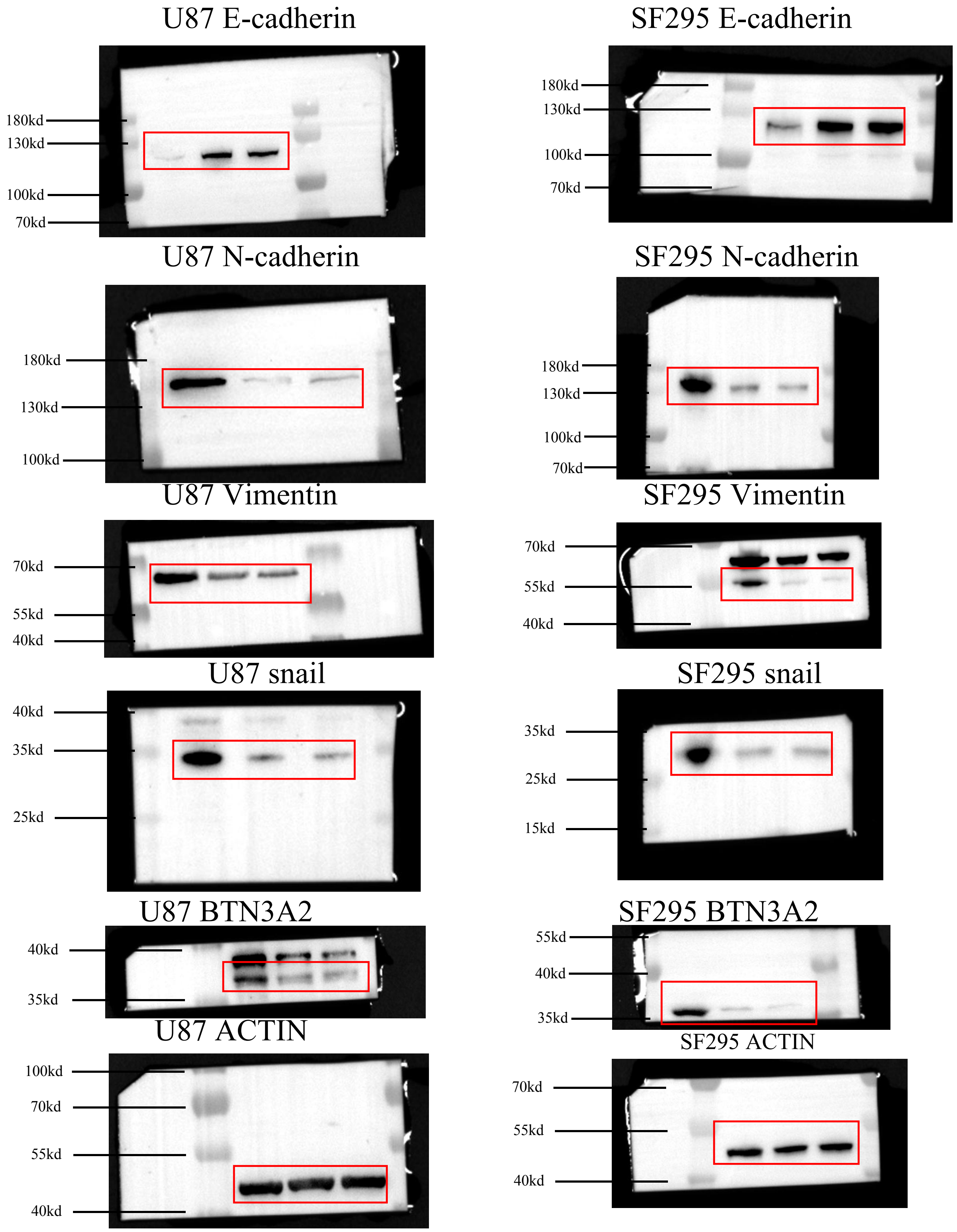
U87 E-cadherin
180kd
130kd
100kd
70kd
SF295 E-cadherin
180kd
130kd
100kd
70kd
U87 N-cadherin
180kd
130kd
100kd
SF295 N-cadherin
180kd
130kd
100kd
70kd
U87 Vimentin
70kd
55kd
40kd
SF295 Vimentin
70kd
55kd
40kd
U87 snail
40kd
35kd
25kd
SF295 snail
35kd
25kd
15kd
U87 BTN3A2
40kd
35kd
SF295 BTN3A2
55kd
40kd
35kd
U87 ACTIN
100kd
70kd
55kd
40kd
SF295 ACTIN
70kd
55kd
40kd

Uncropped blots related to Figure3

Fig.3D

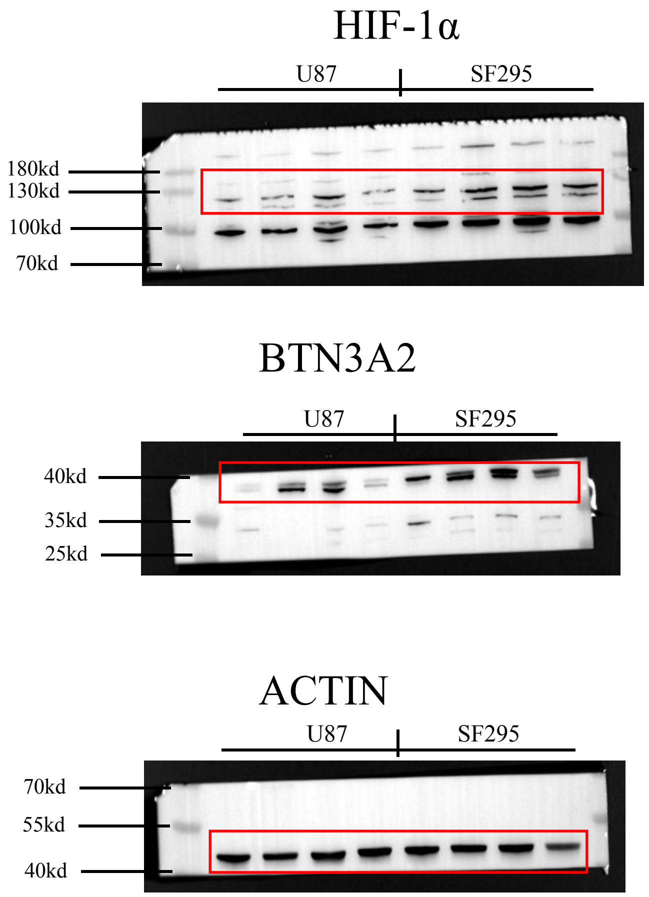

Fig.3F

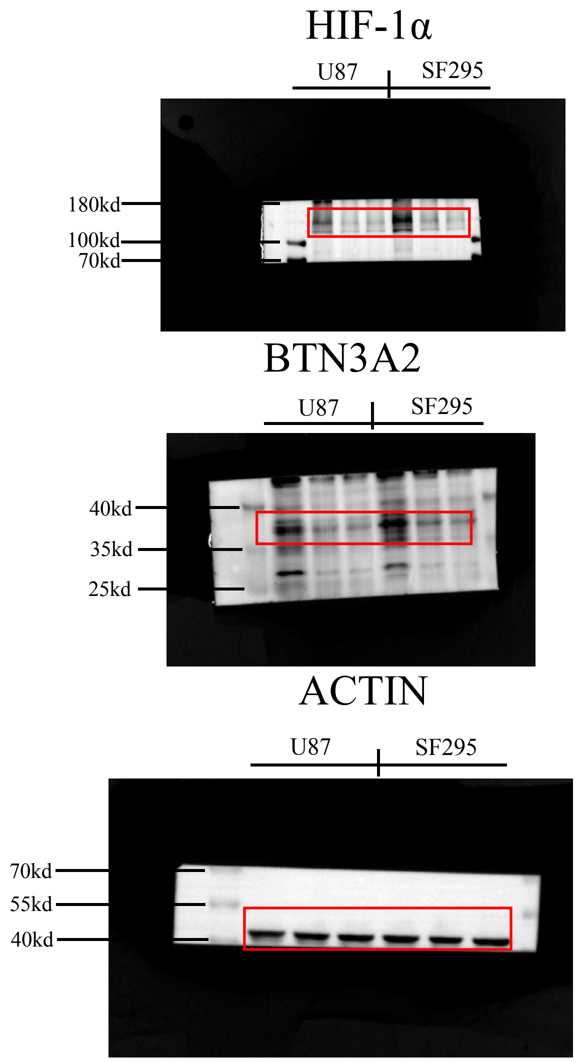

Uncropped blots related to Figure4

Fig.4D

Fig.4F

U87 PARP1

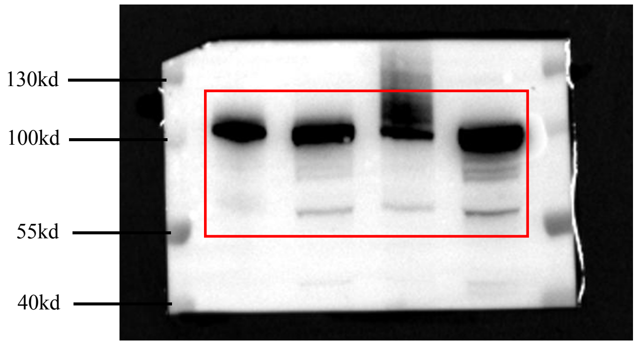

SF295 PAPR1

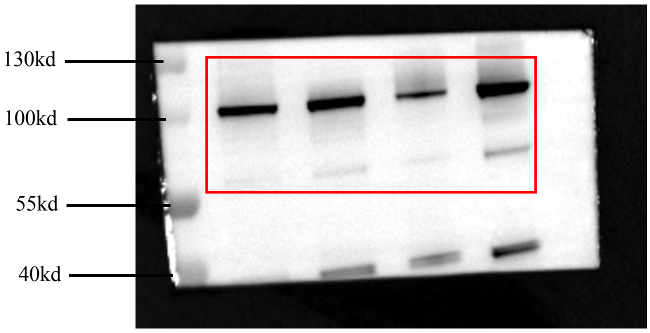

U87 cleaved-casp3

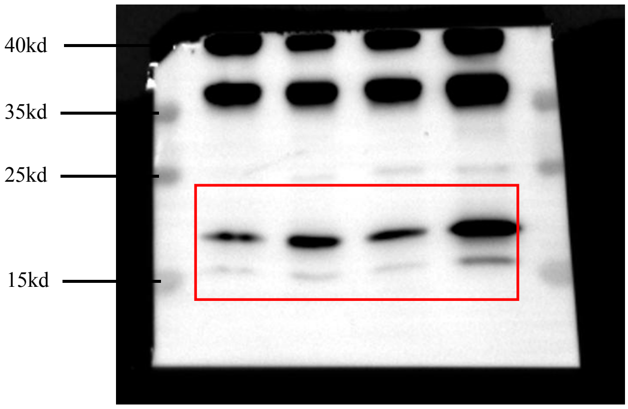

SF295 cleaved-casp3

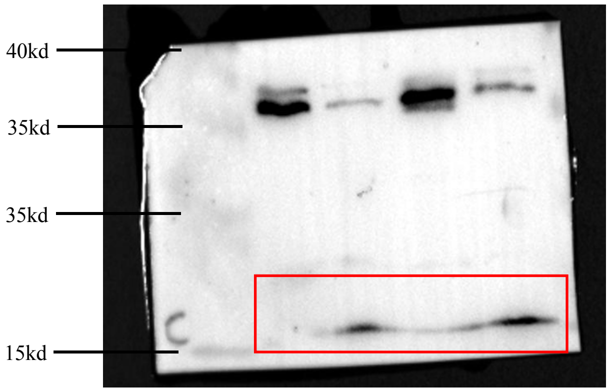

U87 ACTIN

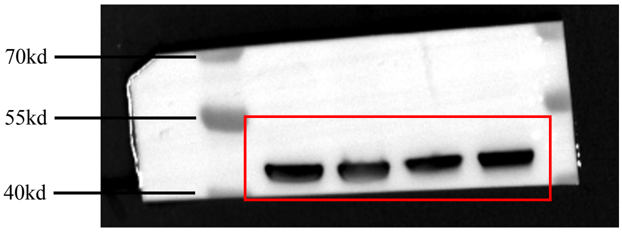

SF295 ACTIN

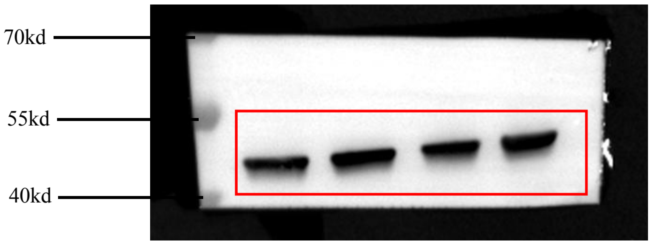

Uncropped blots related to Figure5

Fig.5I

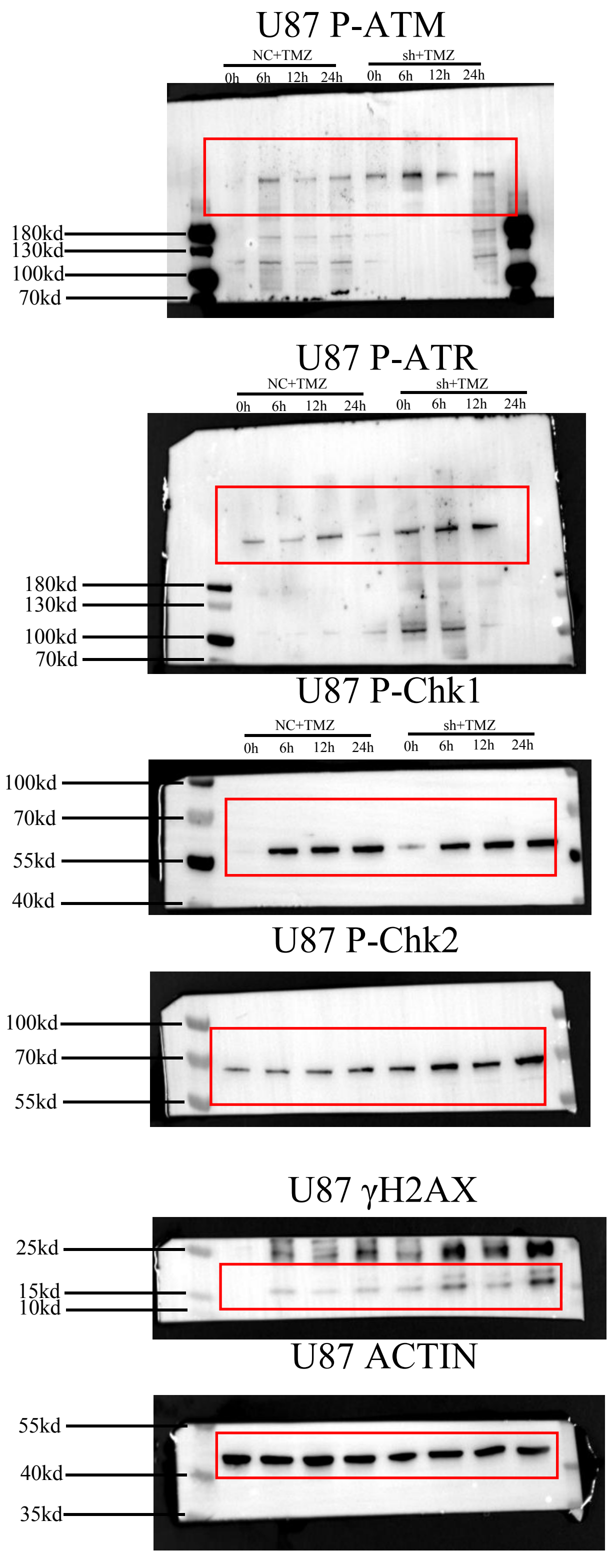

Fig.5J

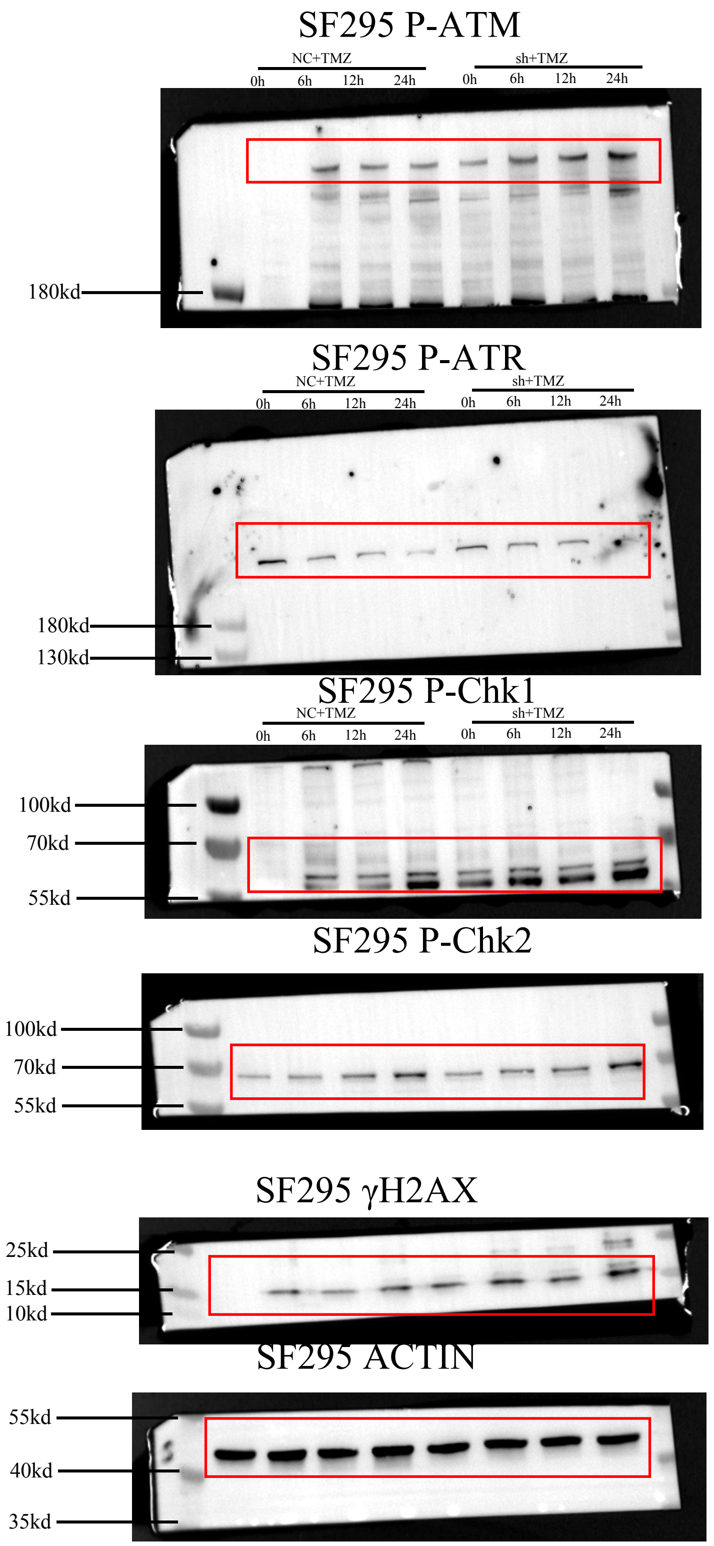

Uncropped blots related to Figure6

Fig.6F

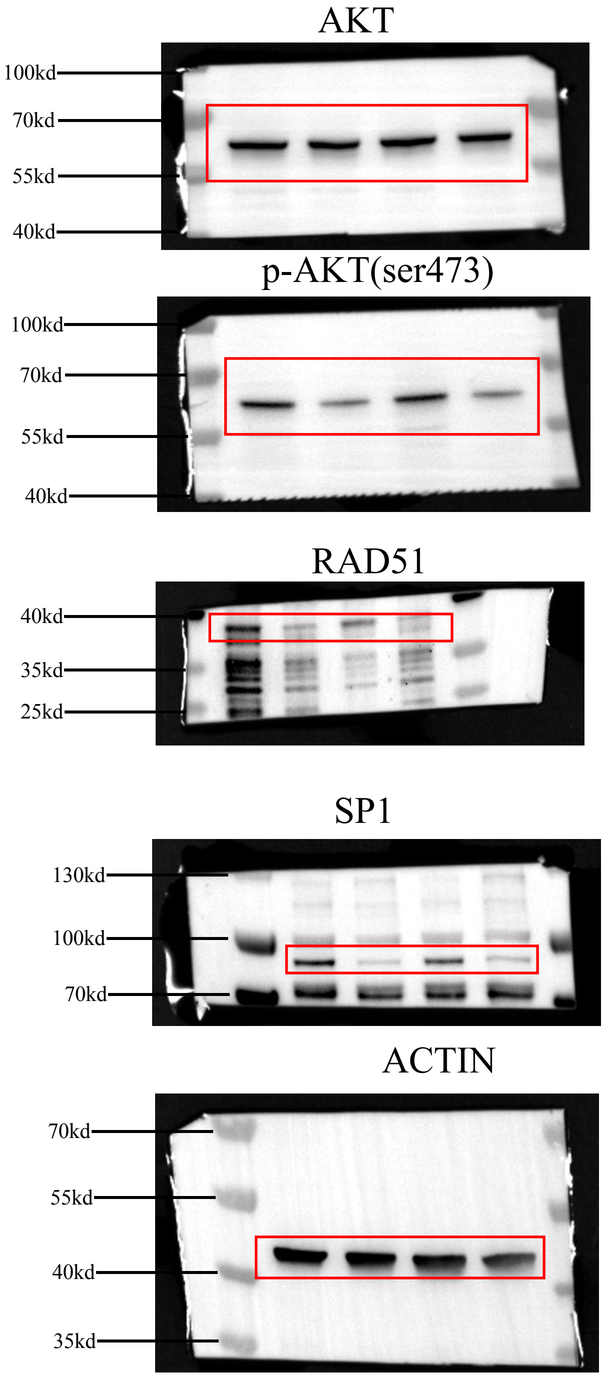

Fig.6I

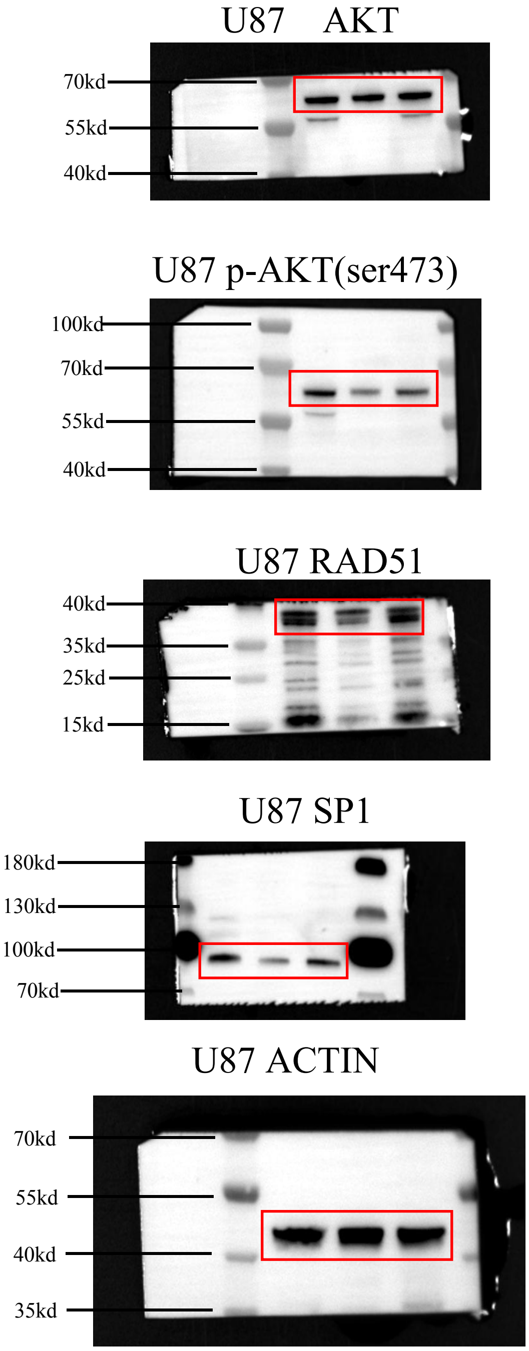

Uncropped blots related to Figure6

Fig.6I

SF295   AKT

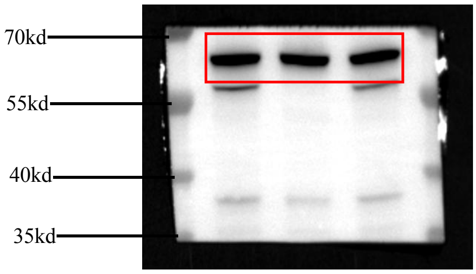

SF295   P-AKT

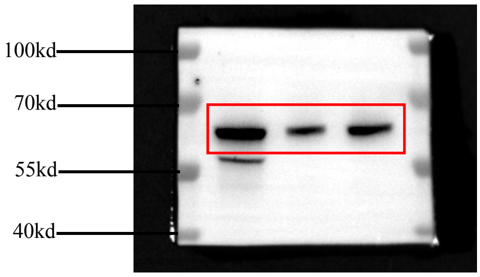

SF295 RAD51

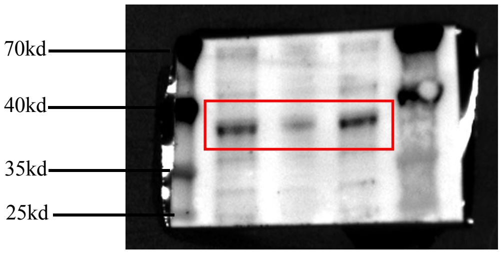

SF295 SP1

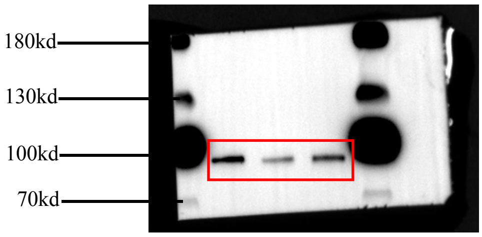

SF295 ACTIN

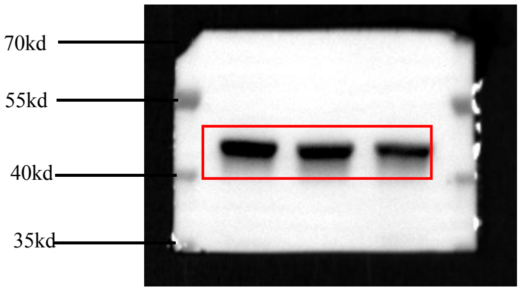

Uncropped blots related to FigureS2

FigureS2.G

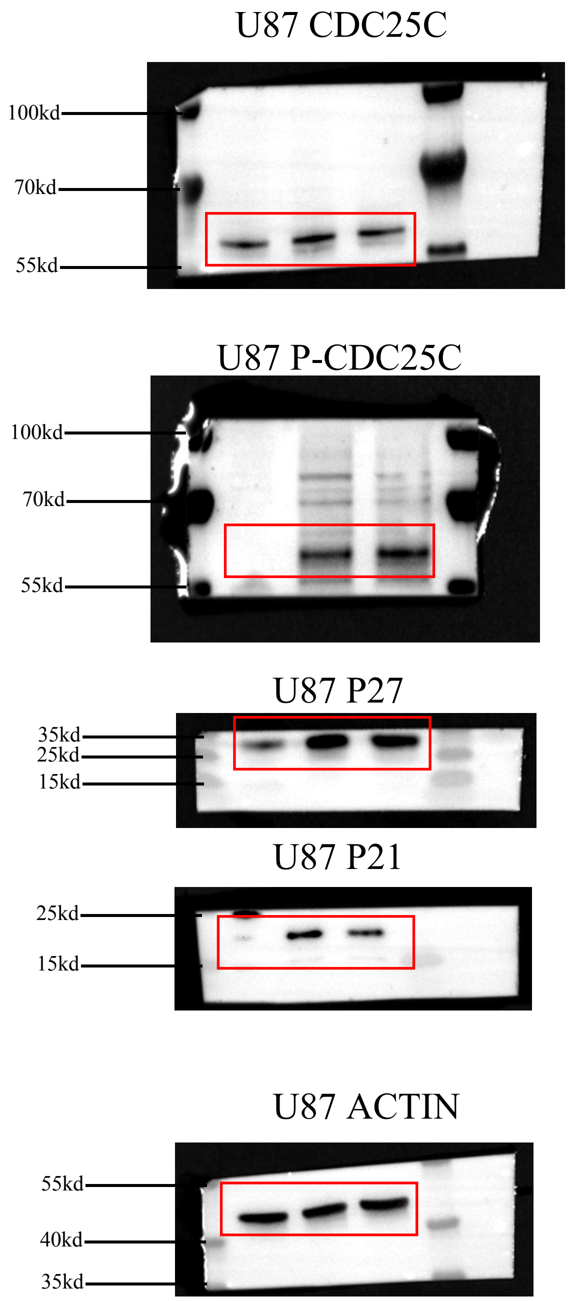

FigureS2.I

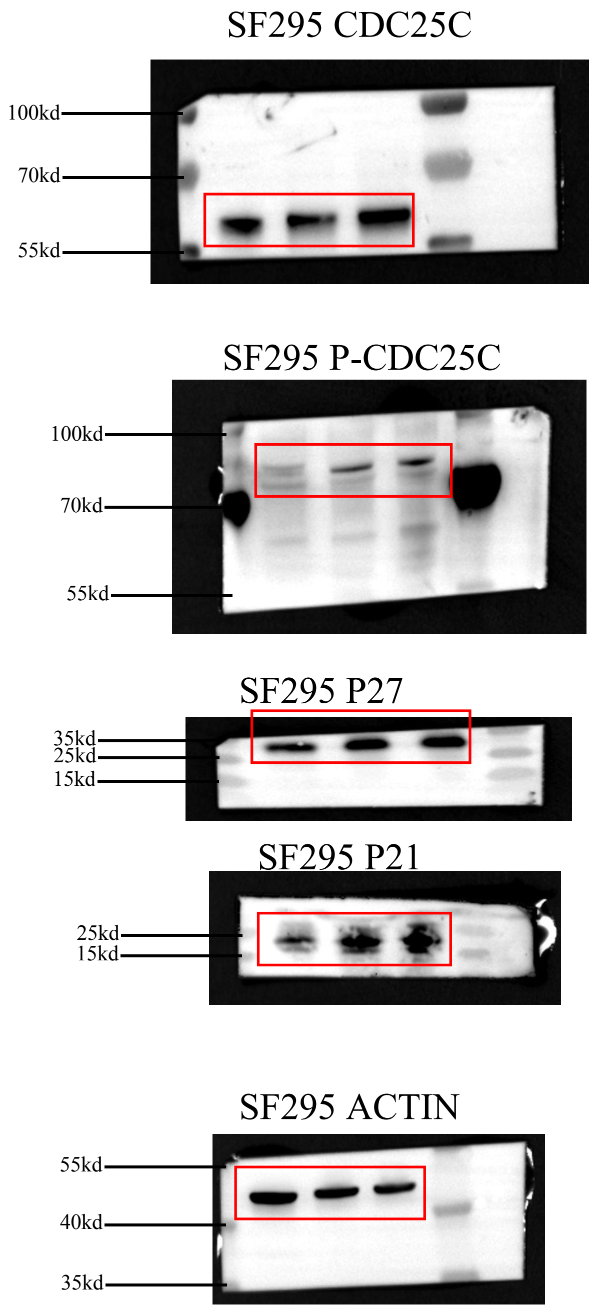

Supplement: Supplementary file 3 — FigureS1 [file 41419_2026_8729_MOESM3_ESM.pdf]
