# Supplementary figures and images for: Hypoxia-induced BTN3A2 promotes glioma progression and chemoresistance via AKT/SP1/RAD51-mediated DNA damage
Source: Cell Death Dis. 2026 Apr 11;17(1):469. doi: 10.1038/s41419-026-08729-7 (PMC13181034; doi:10.1038/s41419-026-08729-7)

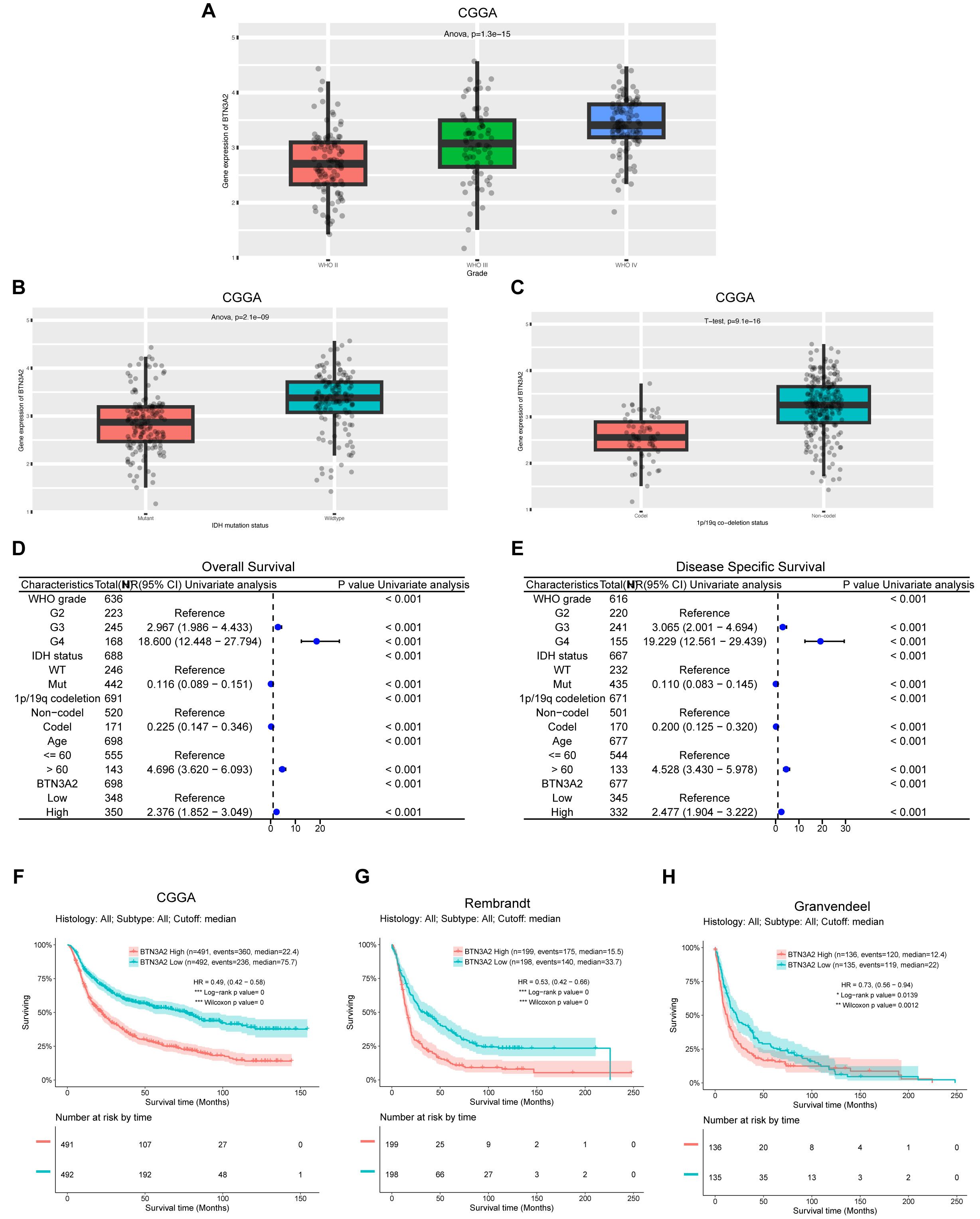

Supplement: Supplementary file 4 — FigureS2 [file 41419_2026_8729_MOESM4_ESM.tif]

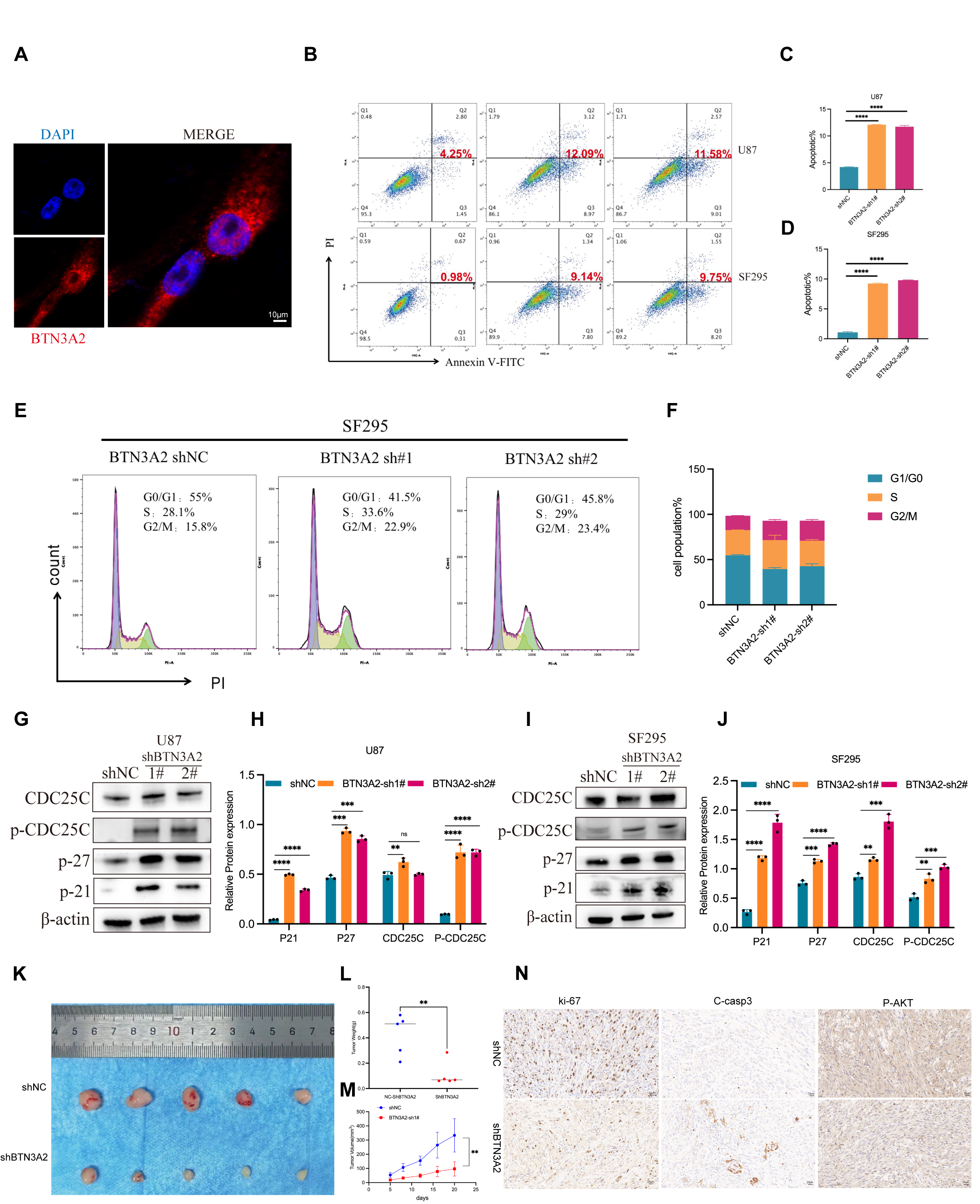

Supplement: Supplementary file 5 — FigureS3 [file 41419_2026_8729_MOESM5_ESM.tif]

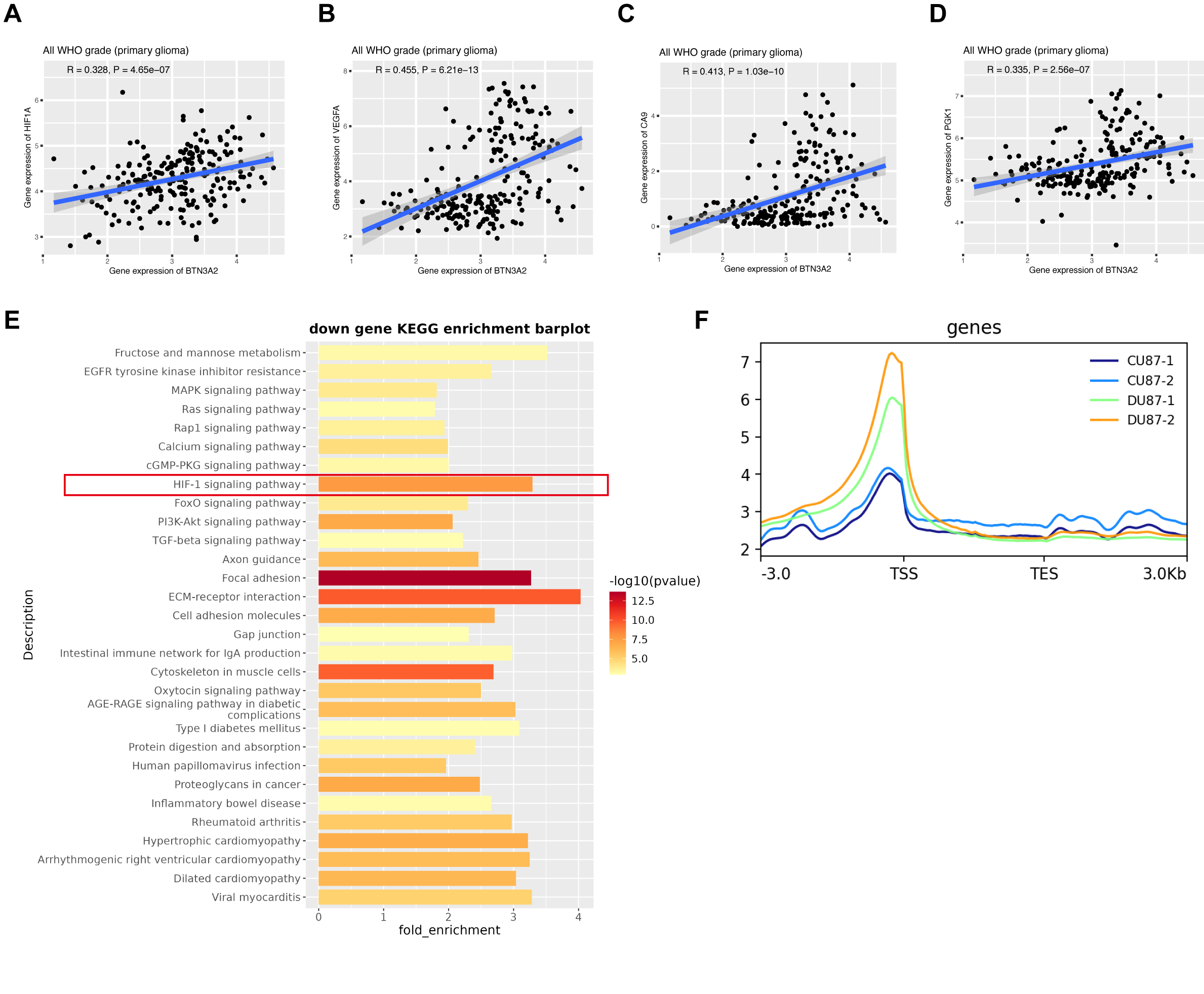

Supplement: Supplementary file 6 — FigureS4 [file 41419_2026_8729_MOESM6_ESM.tif]

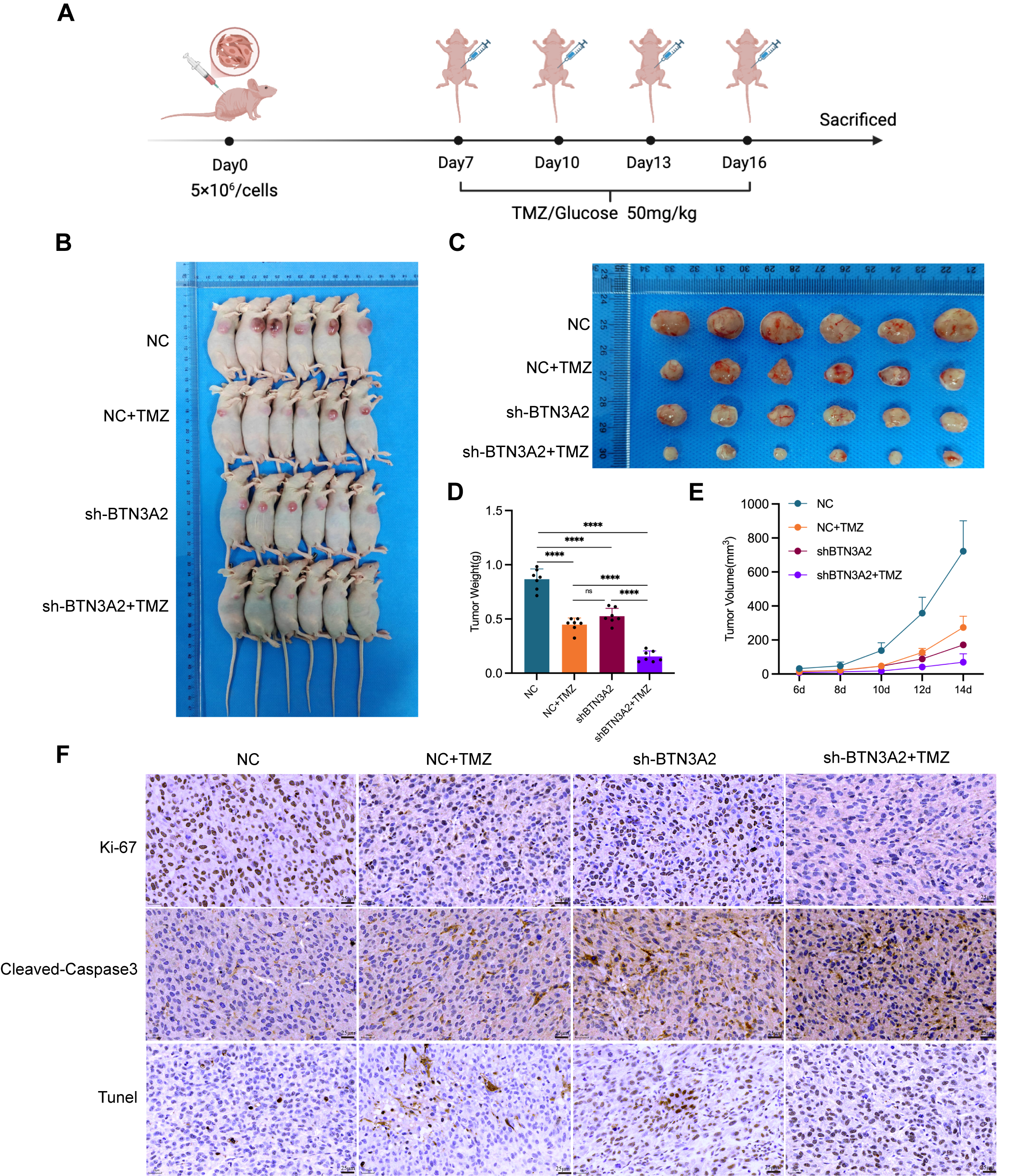

Supplement: Supplementary file 7 — FigureS5 [file 41419_2026_8729_MOESM7_ESM.tif]

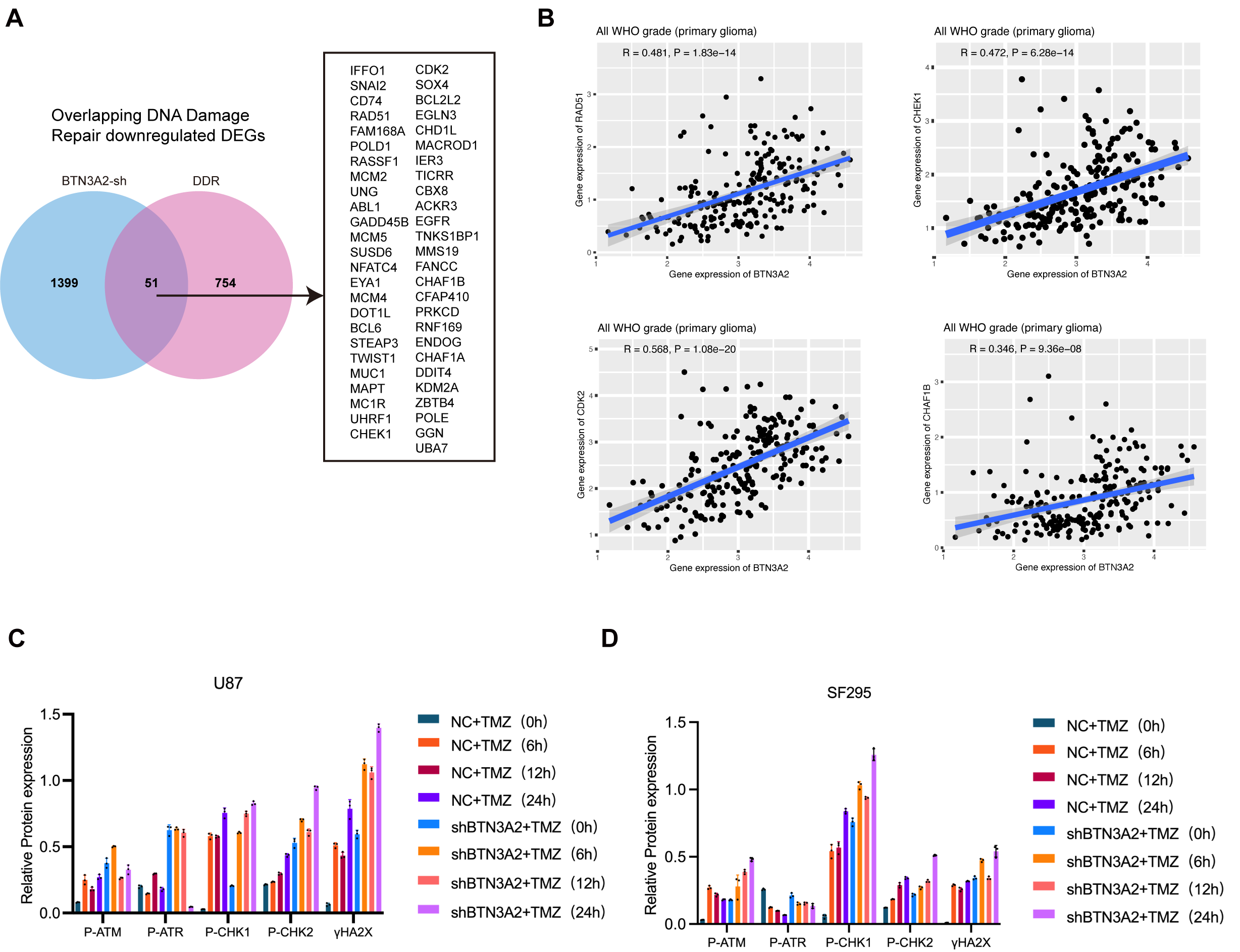

Supplement: Supplementary file 8 — TableS1 [file 41419_2026_8729_MOESM8_ESM.tif]
